# Self-Concentration Detection Based on Doubled Amplitude/Phase Processing in Node PDE Modular Models

**DOI:** 10.3390/biomimetics11090681

**Published:** 2026-09-21

**Authors:** Ladislav Zjavka

**Affiliations:** Department of Computer Science, Faculty of Electrical Engineering and Computer Science, VŠB-Technical University of Ostrava, 17. Listopadu 15/2172, 70800 Ostrava, Czech Republic; ladislav.zjavka@vsb.cz

**Keywords:** personal concentration, deep learning, differential learning, derivative transformation, sine/cosine representation

## Abstract

Reliable classification of brain sequence cases is a challenging problem due to signal ambiguity and noise. Personal concentration is primarily determined by the base frequency of electroencephalogram (EEG) waves, i.e., the task rests on appropriate modelling and recognition of patterns in the corresponding human (in)activity (e.g., reading, relaxation, solving maths problems, etc.). Five underlying types of frequency (alpha, beta, gamma, delta, and theta) were considered as secondary input wave parameters in complex-valued node extensions to the prime amplitude in processing signals. Self-optimisable Artificial Intelligence (AI) methods can process, statistically analyse, and model the series-specific character and time behaviour to recognise untrained session assigned labels. This procedure involves signal pre-processing (transformation) and feature extraction to enhance the representation in time variability, eliminate uncertain cases, and reduce the unacceptable large raw format of data in detailed frequency band recording. This study focuses on improving AI modelling through brain-inspired doubled amplitude/frequency signal processing. This extended concept is based on an analogy with neural activity that generates dynamic frequency pulses as the main information holder in response to time excitations. The model is obtained in partial differential equation (PDE) solutions of evolutionary tree structure nodes—self-computational terms, using the optimal sine/cosine or rational expression. It enables the representation of periodic patterns in their intrinsic form related to primary wave characteristics. Two different machine learning methodologies were compared: the first evolutionary PDE transform and deep learning-based recurrent processing applied to all session records in bloc, assessing only one final class assessment, achieving predictive accuracy above 90% on untrained 1/3 data. The second group of regular modelling techniques evaluates each data row separately to compute its bound-label output in a time-lagged frame, reaching accuracy above 70%. An executable parametric software with a link to the public EEG data repository is available.

## 1. Introduction

Human electroencephalogram (EEG) signals provide fundamental information, inevitable in non-invasive recognition and modelling of various emotional mind states (relaxation, solving tasks, reading, imagining, etc.). EEG temporal signals recorded for well-marked human emotion states seem to exhibit very similar oscillation behaviour, maybe scatterbrained and labelled into incorrect categories, which essentially limits the model generalisation across various specifics of subjects. The period and shape of brain waves seem to provide the most essential information on characteristic mental patterns directly related to electrical activity in specific neural centres. Automatic detection of human cognitive activity is essential to assess personal condition, performance, or sleep stages in military, health, safety, or education applications based on affective computing [1]. Detecting mental states according to base-frequency periods is a difficult task due to the large individual variability in cognitive events in discriminating feature evaluation. This problem can be seen as the monitoring of different parts of brain centres responsible for specific activities in combination with local EEG activations and functional personal connectivity [2]. The brain–computer interface (BCI) is an interactive platform that transforms neural activation into commands for external devices. It plays an important role in communication with immobile patients or disabled people, suffering from hard disease or damage to the motion apparatus; rehabilitation; monitoring activities in unsafe, hazardous, or aviatic professions; etc. EEG signal analysis and recognition are necessary to diagnose various stages of mental disability that challenge the precise interpretation of complex EEG patterns [3]. Negative emotions (depression or phobia) essentially influence the physical and mental being [4]. Their recognition is of practical importance in the diagnosis of various neurodegenerative or psychiatric disorders [5].

Personal concentration is a state of increased neurophysiological activation characterised by the intended alignment of cognitive attention in practising specific tasks by suppressing external and internal perturbations. The level of concentration represents a high-/low-intensity mental state of sustained selective attention. In real-world environments, cognitive concentration is a highly dynamic, fluctuating, resource-heavy mind frame where brain activity corresponds to amplified relevant sensory inputs, suppressing distraction noise. These personal states are characterised by specific changes in the nervous, cardiovascular, and metabolic systems. A laboratory apparatus measures attention as a stable controlled variable, although the brain is constantly forced to negotiate demands between internal and external stimuli in the parallel process. Self-labelled scores can correspond (moderate to strongly) to established cognitive load metrics, but the relationship depends heavily on what the user evaluates and how this is administered. Self-labelled ratings are highly correlated with behavioural metrics measured as the functional output of cognitive load in controlled settings. However, their decoupling is usually closely dependent on a variety of individual skills. Although a binary recognition model (1.0 for mental arithmetic versus 0.0 for the rest) offers an exact baseline, real human concentration is naturally more complex and fluctuating. Self-reported scores capture an individual’s conscious perception of mental effort compared to standard objective cognitive load metrics generally related to multidimensional frameworks. Specific neural biomarkers (such as the theta/beta ratio) can be used in statistical modelling to map continuous subjective scores to objective concentration levels. Objective EEG metrics can correct subjective perception with respect to defined neural schemes in evaluated mental tasks [6].

A new designed differential learning (DfL) uses standard amplitude and extra-frequency EEG signal data in the double form of pair-matched node inputs. Its modular self-evolving architecture, resembling self-organising neural structures, produces partly autonomous computing components as node-determined sum PDE periodic/rational solutions. This novel concept extends the regular single-valued amplitude data processing with bound frequency characteristics, analogous to brain pulse operation dynamics, better reflecting signal periodicity in the intrinsic phase form. Neurone-like composite sum terms produced in node-by-node evolving binomial tree structures are self-contained adaptable/selective units computing the model overall separable output.

Deep learning techniques have recently emerged in the field of biomedical engineering and have successfully demonstrated their effectiveness in classification using recurrent processing of large data series [7]. They obtain higher predictive accuracy compared to conventional techniques, based on estimating a label output for each data record separately. However, the Long Short-Term Memory (LSTM) cell-state approach usually suffers from long-range time series model dependence and computational ambiguity, in addition to limited development capability in the temporal, frequency, and spatial domains. Integrating features from the temporal, frequency, and spatial domains attempts to overcome some shortcomings. This study evaluates two distinct types of key methodologies in pattern identification.

- The first sequence-to-all-session approach compares the PDE transform component models, produced by binomial tree structures, and the recurrent LSTM cell processing of sequenced input. A final prediction corresponds to percentage statistics in the successful detection of EEG row patterns or internal memory states, comprising the same label (one output for the entire session).
- The second type of sequence-to-sequence classifier assigns the output for each individual EEG data row label in relation to a lagged input from previous series. Each session row is scored from the output <0, 1> output in class ‘0’/‘1’ to be evaluated in the final percentage precision.

Various types of recent machine learning (ML) classifiers (E. MATLAB—Deep Learning neural network for Classification (DLC) https://www.mathworks.com/help/deeplearning/ug/deep-learning-in-matlab.html, www.mathworks.com/help/deeplearning/ug/classify-sequence-data-using-lstm-networks.html URL (accessed on 18 September 2026). Classify ECG Signals Using Long Short-Term Memory Networks https://www.mathworks.com/help/deeplearning/ug/classify-ecg-signals-using-long-short-term-memory-networks.html URL (accessed on 18 September 2026)) (F. MATLAB—Classification Learner App (CLA) https://www.mathworks.com/help/stats/classificationlearner-app.html URL (accessed on 18 September 2026). Choose Classifier Options www.mathworks.com/help/stats/choose-a-classifier.html URL (accessed on 18 September 2026). Ensemble algorithms www.mathworks.com/help/stats/ensemble-algorithms.html URL (accessed on September 18, 2026)) (G. Shallow Neural Network (SNN) https://www.mathworks.com/help/deeplearning/ref/network.train.html URL (accessed on 18 September 2026). Improve Shallow Neural Network Generalisation and Avoid Overfitting https://www.mathworks.com/help/stats/fitcnet.html URL (accessed on 18 September 2026)) along with analytical methods were examined in EEG mind state detection:

◦ Differential computing network;
◦ Recurrent LSTM network;
◦ Shallowed/neural network;
◦ Ensemble bagged/boosted tree;
◦ Gaussian process regression, Naive Bayes;
◦ Support Vector regression;
◦ Logistic regression, Discriminant Analysis, Nearest Neighbour, etc.

Differential Computing Network (DCN) is a novel hybrid neuro-adaptive/algebraic-based technique that embodies DfL (designed and developed by the author). It combines ML principles in a step-by-step evolved tree structure with customised numerical procedures of operator calculus (OC) to solve node-defined PDEs. The concept of DCN was extended to double processing of the amplitude/phase data in a matched form; that is, the two input pairs of each node are combined in the computed output for both signal characteristics. This approach allows for advanced wave representation of the recognised/modulated original frequency input in periodic PDE-transform components formed in DBN nodes. The sum of selected reconverted PDE-node modular solutions gives the overall DCN output. The DfL novelties and incremental enhancements include:

- DCN elicits binomial structures by adding node by node to decompose the *n*-variable general PDE into a set of determined node PDEs using conversions based on OC.
- Applicable sub-PDE sum components are composed of several types of rational, periodic, or power OC substitution functions in nodes to minimise the output error.
- Periodic PDE-node conversion, based on the L-transformation or Fourier time series, allows modelling of periods in ambiguous EEG wave signals.
- The pulse amplitude and phase input pairs are processed in a doubled form, weighted/combined separately in the node output and jointly in applicable sub-PDE solutions.
- Goedel’s incompleteness theorem-based step-by-step model expansion gradually increases its complexity to reach the external-criterion minima.
- Iterative structure refinement, by adding new or removing redundant PDE composite components (in respect of previous layer solutions), is included in the overall sum model.
- It includes thorough comparison with LSTM-based deep and diverse machine learning classifiers in the statistical evaluation of two strategies (one-session-sequence and sequence-to-sequence).

## 2. State of the Art: EEG Brain Activity Detection

EEG is a highly non-stationary/linear type of multichannel signals that are difficult to analyse, model, or interpret in raw form. The wavelet transformation effectively modulates the time–frequency domain in the data to be widely applicable via analytical or ML tools. EEG signals are decomposed into subband components with reduced noise. Feature extraction improves the classification accuracy of ML models, looking for higher correlations between the input and output [8]. An analogous time–frequency transformation is usually performed with a secondary extraction of purposeful features in pre-processing before regular ML. Bandpass or adaptive filtering can remove most objectional internal artefacts outside the desired EEG frequency range. Independent component analysis in the time–frequency domain attempts to separate and evaluate statistically distinct features of mixed EEG signals. It assumes that the raw EEG data come from sources within the maximum of active channels. BCI-based motor imagery can adopt the multifrequency nature of brain signals by combining several band channels in time domains to improve feature extraction. The same approach is used to extract spatiotemporal EEG characteristics in attentional learning to reveal dual internal relations in continuous-time multichannel data [9]. The attentional mechanism can be used to predict an epileptic attack using differential EEG entropy data [10].

Traditional recognition of EEG patterns using neural networks (NNs) employs only a shallow structural framework, usually unable/unreliable to find significant biomarkers in 2-hidden layers. In addition, they suffer from the ineffective handling of large EEG data for all frequency bandwidths. Deep learning (DL) based on the LSTM architecture can reduce this constraint in the detection of progressive neurodegenerative disorders. The complete modelling procedure includes denoising, segmentation, downsampling, and uncovering hidden deep-delay features [11]. EEG data typically need to be collected from many individual-labelled measurements comprising different types of subjects to avoid the multivariate character, as subject-specific data suffer from generalisability. Transfer learning and pre-training can be used by integrating training samples from multiple subjects [12]. Matrix learning takes advantage of structural information between columns and/or rows in feature records. Neural pattern decoding overcomes this problem using an adaptive transfer matrix built from multiple sources to enable modelling of structural information from the corresponding EEG feature matrices [13]. Deep learning-based emotional state recognition can employ effective connectivity maps to measure the flow of EEG information. Transfer entropy and coherence functions are used to produce a fused image processed as a classification input to improve accuracy [14]. Frequency EEG band biomarkers capture neural activity with connectivity patterns directly related to cognitive abilities that are crucial in the identification of neurodegenerative diseases. To detect them correctly, EEG signals are first denoised and segmented into continuous-time sequences. Subsequently, important frequency subbands are extracted and combined from each time segment to generate spectrograms, that is, images applied as input from the model [15].

EEG signals corresponding to several emotional states have similar oscillations in their nature. Data are often aligned with incorrect or confused categories, which limits their generalisability in modelling across subjects. The large variability and subject-independent emotion complicate the evaluation process [16]. Domain-adaptive methods can compensate for the variability in the characteristics of different subjects [17]. Detecting several levels of ever-lasting attention in the non-stop operations is important in real-world applications to avoid fatal accidents or collision consequences resulting from reduced attention. The frontal complexity of dynamic EEG patterns appears to be correlated with the response action in continuous-attention tasks [18]. Monitoring the depth of anaesthesia improves the evaluation of low-wave EEG levels in clinical surgery through cerebral electrical activities. Consciousness sleep states can be detected using a deep metalearning LSTM framework, combined with a graph convolutional NN [19], capturing temporal correlations in data for a high diversity of subjects, using pre-identified features in the power spectrum [20]. Unconventional approaches to short-term emotion detection based on a spiking NN allow dynamical modelling of spatiotemporal EEG patterns [21]. Fractal functions can generate modified EEG data from original features to improve the recognition of emotional patterns [22].

DCN applies L-transformation to node-detected PDE-types, which improves the trainability of data in EEG pattern variability. It performs automatic self-extraction of input pairs to evaluate each individual node-selected component that is consequently included in the extended sum model. The periodic PDE representation enables capturing pulse dynamics and time variability in non-stationary frequency signals. The rational PDE or power-transform components reflect a degree of non-linearity in amplitudes and wave distortions. The large modularity and diversity in backward node-produced composite terms (rational, periodic, power) enable reducing the ambiguity and uncertainty in the modelling of EEG patterns. DCN models are produced by adding node-by-node in a binomial structure, using three-level iterative self-optimisation in synchronising: 2-node input selection, PDE node-type detection, and parameter gradient adaptation. The combinatorial PDE component models are composed and fitted step by step to reflect specifics in time variability dynamics and complexity in EEG patterns.

## 3. Acquisition of EEG Data Using the 4-Channel Muse 2 Headband

EEG data are usually measured in a large data format of standardised bandwidths. Downsampling is applied first to reduce the unacceptable size of raw series and enable effective use. Referencing compares the electrical activity recorded by an EEG electrode with the voltage (mean) recorded by other electrodes or bias. Internal noising can arise from ocular movement, muscle action, heart beating, respiration, etc., combined with additional external error sources (e.g., wiring, electrode connections, power supply). Subjects can be asked to perform a task, invoke imagination or a mental state, and be exposed to a variety of media types: sights, sounds, videos, or other secondary sensory input [23].

EEG signals are generally categorised into a few main frequency bandwidths (Table 1) in considering small variances in the determining range: delta (0.5–4 Hz), theta (4–7 Hz), alpha (8–12 Hz), beta (16–31 Hz) and gamma (36–90 Hz). EEG signals are automatically generated; subjects do not have control over their propagation through neural activity, which is a basic issue in the recognition of emotional patterns [8]. People cannot convey or reveal electrical activity of the brain centres influenced by their psychological and health condition, thoughts, state of mind, other human beings, etc.

**Figure 1:**
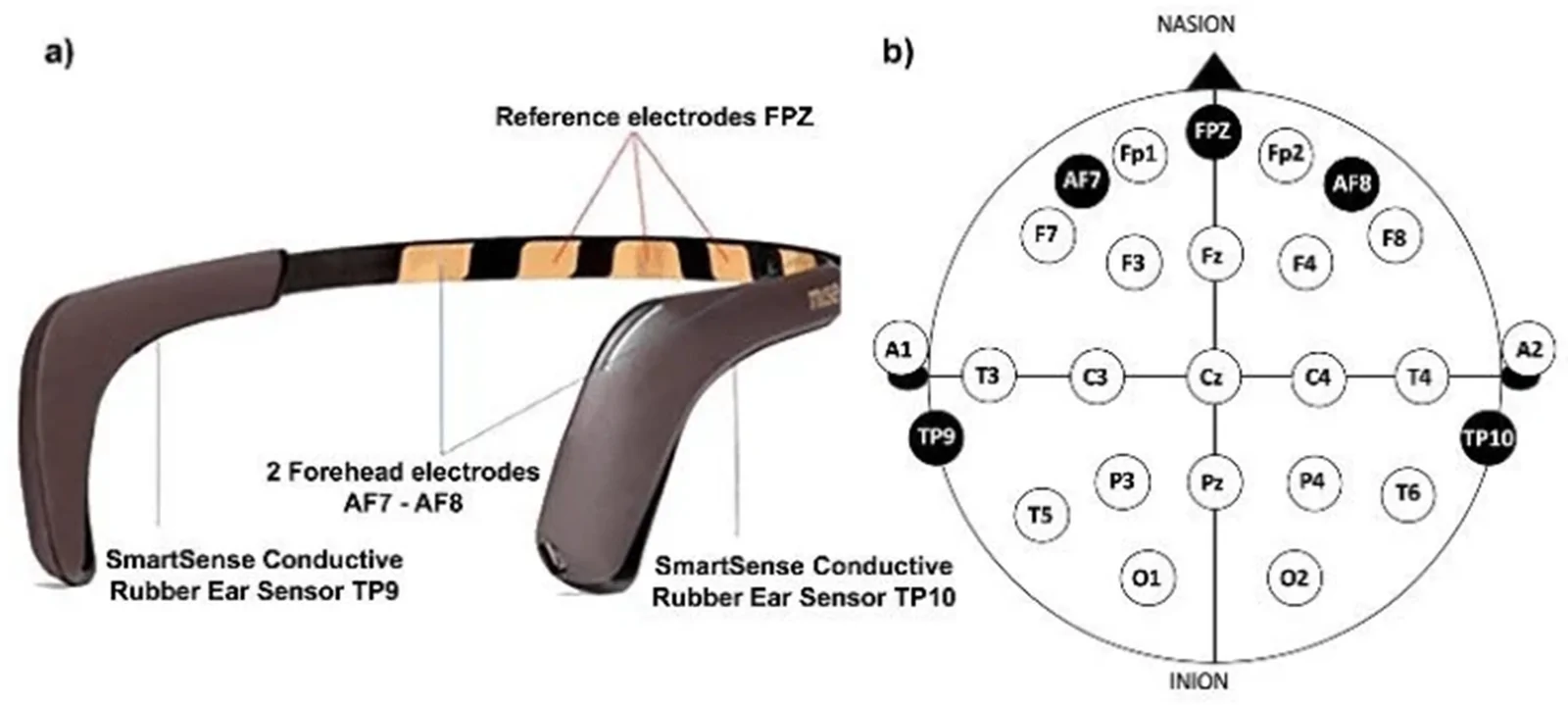
Muse 2 headband sensors (**a**) with standardised top–down positioning of the EEG electrodes (**b**).

The Muse 2 home headband (Figure 1a) was used for personal concentration EEG data recordings for several months. The laboratory subject was male and 20–21 years old at the time of the experiments (A. Muse 3-channel EEG headset https://choosemuse.com/pages/science (accessed on 18 September 2026)). Monitored/performed tasks were as follows:

◦ Mental arithmetic (high concentration level 1.0).
◦ Reading technical articles (medium–high concentration).
◦ Listening to technical podcasts, reading the transcript (medium–high concentration).
◦ Browsing the internet (low–medium concentration).
◦ Just sitting, eyes open or closed, relaxing (low concentration level 0.0).

**Figure 2:**
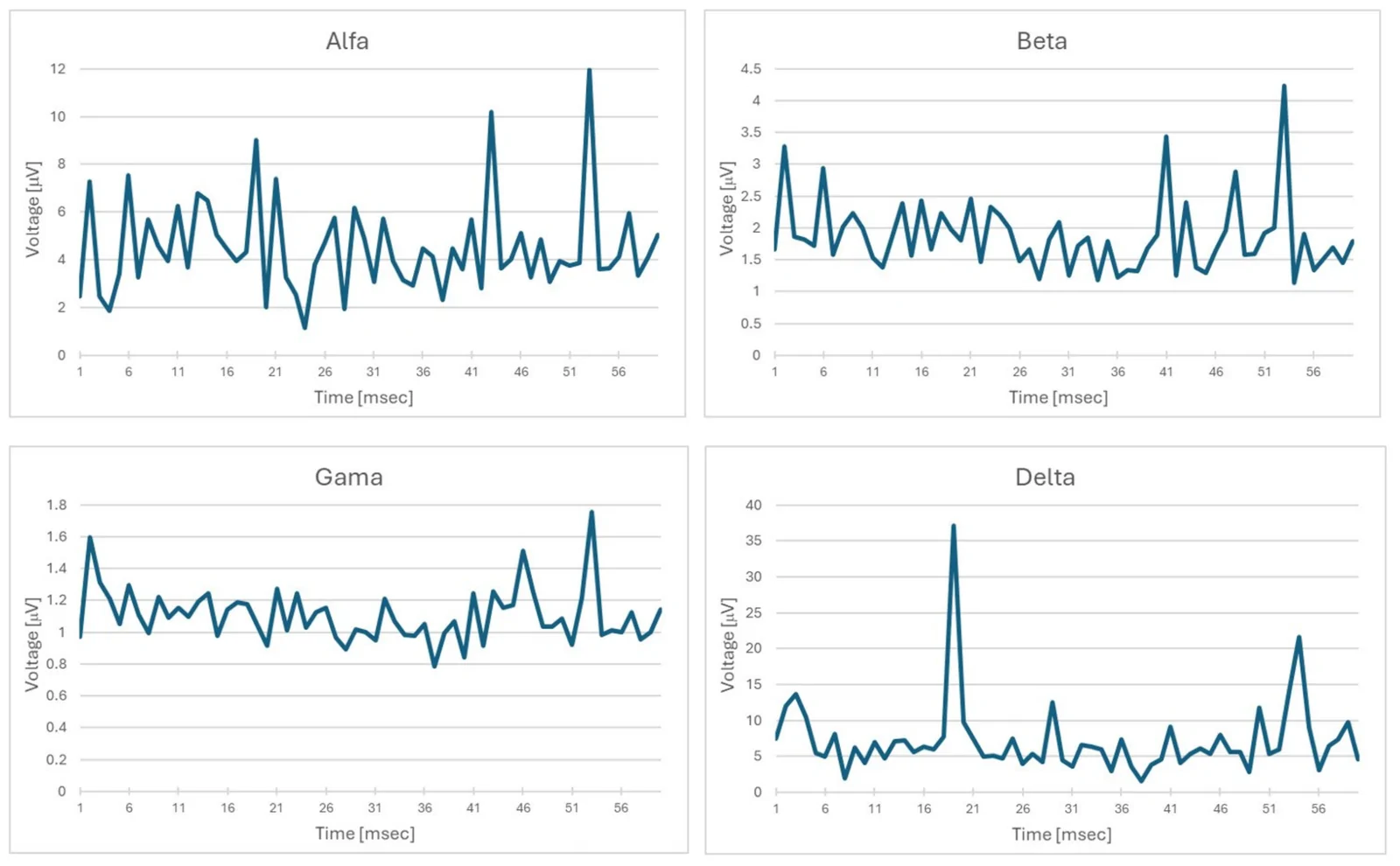
Examples of base-frequency bands in EEG data transformed with FFT recorded from the TP9 zero channel in the first 30 [msec], i.e., 60 rows (concentration level 0.7).

The Muse 2 EEG headband allows for brain wave tracking at a 256 Hz sample rate and 12-bit sample depth with integrated data processing, analysis, and interpretation tools. The recorded data comprise five wave bands: alpha, beta, gamma, delta and theta, passively measured on four silver electrodes (TP9, AF7, AF8, TP10, and reference Fpz; Figure 1b) in micro voltage intensity [μV] (see Muse 2 specification [A]; Table 2, Figure 2). The Fast Fourier Transform (FFT), built into Muse software, was first applied to rough signals (128 bins) before recording. The Fourier transform is a mathematical definition that enables one to convert signals sampled in time or space to the same type of output but sampled in the temporal or spatial frequency domain. Fourier pre-processing can reveal important characteristics in row signals, especially frequency components (B. Fast Fourier Transformation https://www.mathworks.com/discovery/fft.html?s_tid=srchtitle_support_results_3_Fast%2520Fourier%2520transform (accessed on 18 September 2026). Fourier transforms www.mathworks.com/help/matlab/math/fourier-transforms.html). FFT is usually applied to sections of EEG series to determine the power distribution in the main frequency bands. The resulting waveforms can be converted into a brain map that displays the power scalp distribution of each wave band. Another method used to analyse EEG signals is power spectrum analysis (PSA) [24]. The Muse 2 is designed for personal use and scientific experiments and data measurement (A. Muse 3-channel EEG headset https://choosemuse.com/pages/science (accessed on 18 September 2026)).

Each data row has a self-assigned concentration value ranking from 0 to 1, including higher scores for mental arithmetic and technical reading and lower scores for relaxing or browsing. The original data repository contains 80 Excel files, one for each hand-labelled session (with the same activity score). The merged EEG data set includes all 7243 rows in 69 subsequent recording sessions with 4 × 5 + 2 = 22 columns (two for time and class label) (C. Personal concentration data sets (80 .CSV files) https://www.kaggle.com/datasets/dqmonn/personal-eeg-tasks (accessed on 18 September 2026)):

Time stamp, Alpha 0–3, Beta 0–3, Gama 0–3, Delta 0–3, Theta 0–3, Concentration (class label).

Some of the 80 separate successive sessions with the same activity score ranged in <0, 1> were joined to the merged two-class file re-labelled with ‘0’ and ‘1’ (C. Personal concentration data sets (80 .CSV files) https://www.kaggle.com/datasets/dqmonn/personal-eeg-tasks (accessed on 18 September 2026)). Table 3 outlines the summary distributions of the concentration positive ‘1’ and negative ‘0’ classes in the 50–50%, 2/3-1/3 portioned (training prediction) and the entire data set. Occurrence in the 50–50% training set, ‘0’: 2266, ‘1’: 1355 (that is, 3621 rows), and the 50% prediction set ‘0’: 1644 and ‘1’: 1977 (3621). In the 2/3-1/3 data in the 2/3 training, ‘0’: 3147, ‘1’: 1681 (4828 rows), and the prediction 1/3 ‘0’: 763, ‘1’: 1651 (2414).

**Figure 3:**
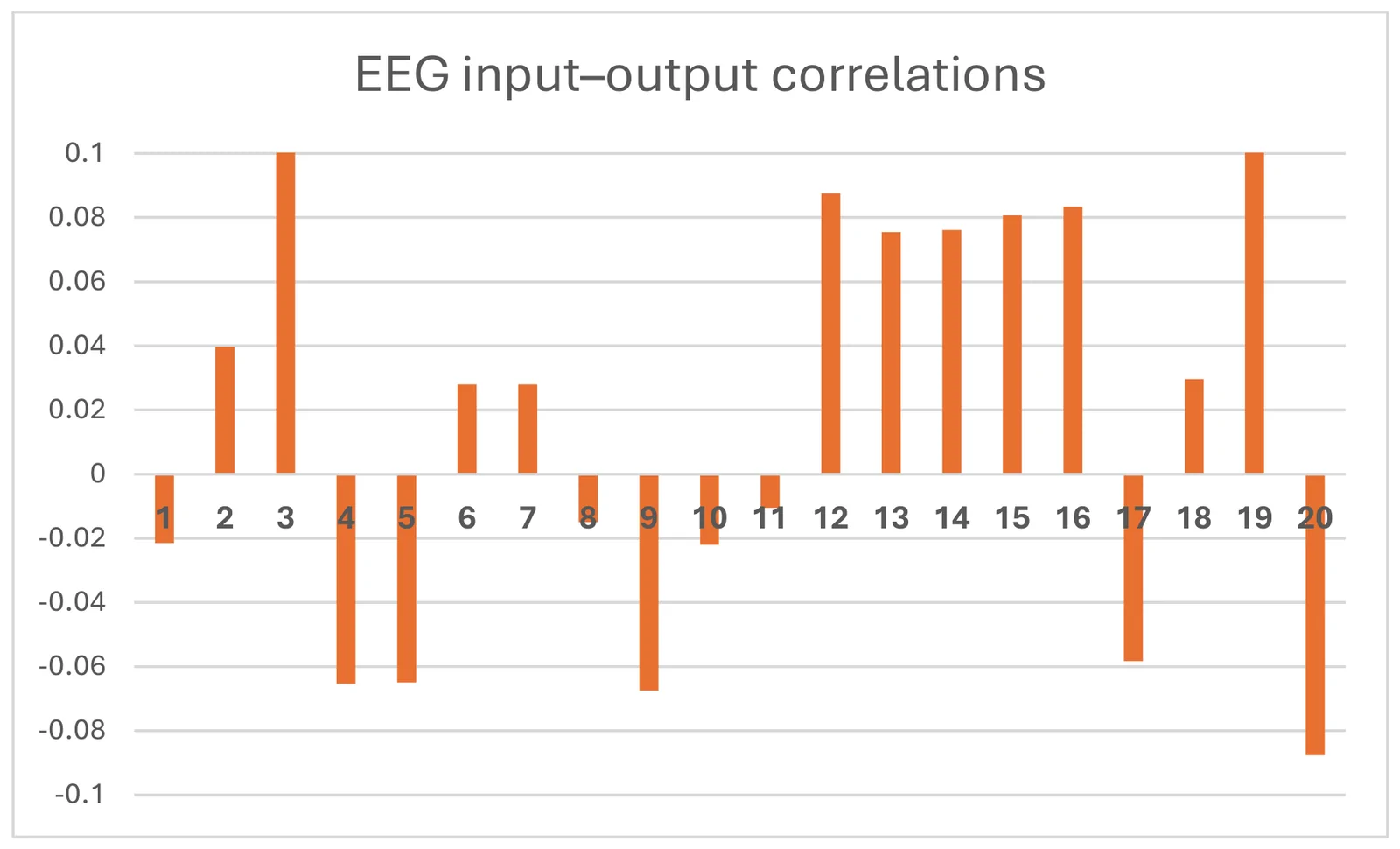
Correlation analysis between 20 EEG input signals and the label <0, …, 1> ranged output (4 sensors TP9, AF7, AF8, TP10: 1 = alpha0, 2 = beta0, 3 = gamma0, 4 = delta0, 5 = theta0, etc.) (C. Personal concentration data sets (80 .CSV files) https://www.kaggle.com/datasets/dqmonn/personal-eeg-tasks) (H. DCN C++ parametric software with one merged Excel EEG data set: https://drive.google.com/drive/folders/1ZAw8KcvDEDM-i7ifVe_hDoS35nI64-Fh?usp=sharing. https://nextcloud.vsb.cz/s/EFDJ7mRyxfHkjSc) (all accessed on 18 September 2026).

Figure 3 shows correlations between the signal EEG input–output <0, 1> original values (H. DCN C++ parametric software with one merged Excel EEG data set: https://drive.google.com/drive/folders/1ZAw8KcvDEDM-i7ifVe_hDoS35nI64-Fh?usp=sharing. https://nextcloud.vsb.cz/s/EFDJ7mRyxfHkjSc). Higher positive or negative values (near +/−1.0) indicate a marked degree of correlation between two input–output quantities. Positive (negative) correlation means that the first column data is positive, and the same holds for those positive in the second column (contrary to the negative, +/− converse data). Values close to zero imply a negligible or marginal correlation level (C. Personal concentration data sets (80 .CSV files) https://www.kaggle.com/datasets/dqmonn/personal-eeg-tasks (all accessed on 18 September 2026)).

## 4. Two Methodologies Applied in Mental States Detection

**Figure 4:**
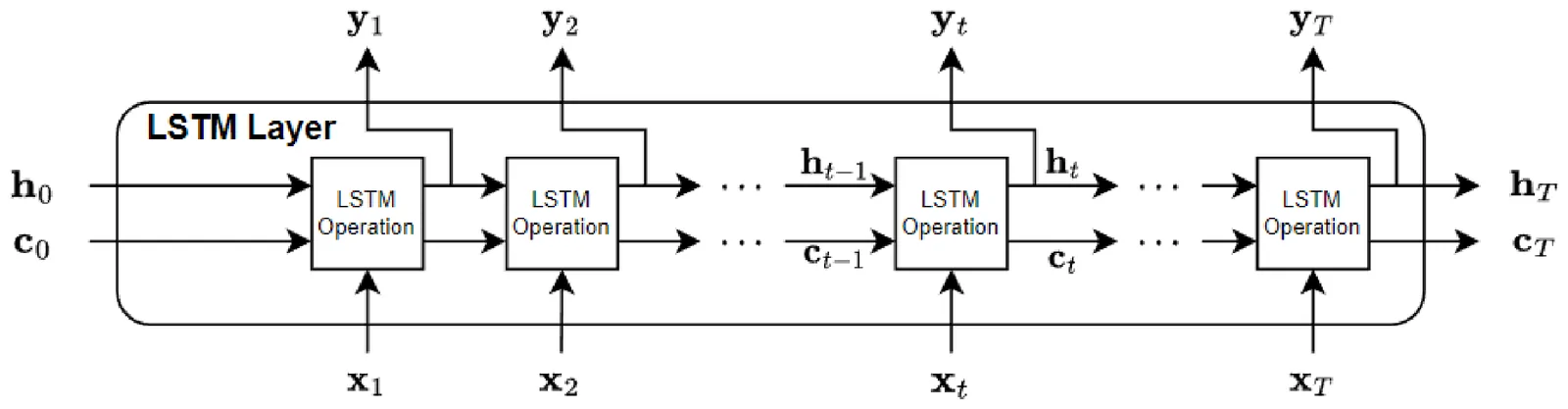
Demonstration of information control (forget or update) in computing output and cell states in the long short-term memory (LSTM) layer (D. Long Short-term Memory (LSTM) network https://www.mathworks.com/discovery/lstm.html?s_tid=srchtitle_support_results_2_LSTM. https://www.mathworks.com/help/deeplearning/ug/long-short-term-memory-networks.html (accessed on 18 September 2026)).

**Figure 5:**
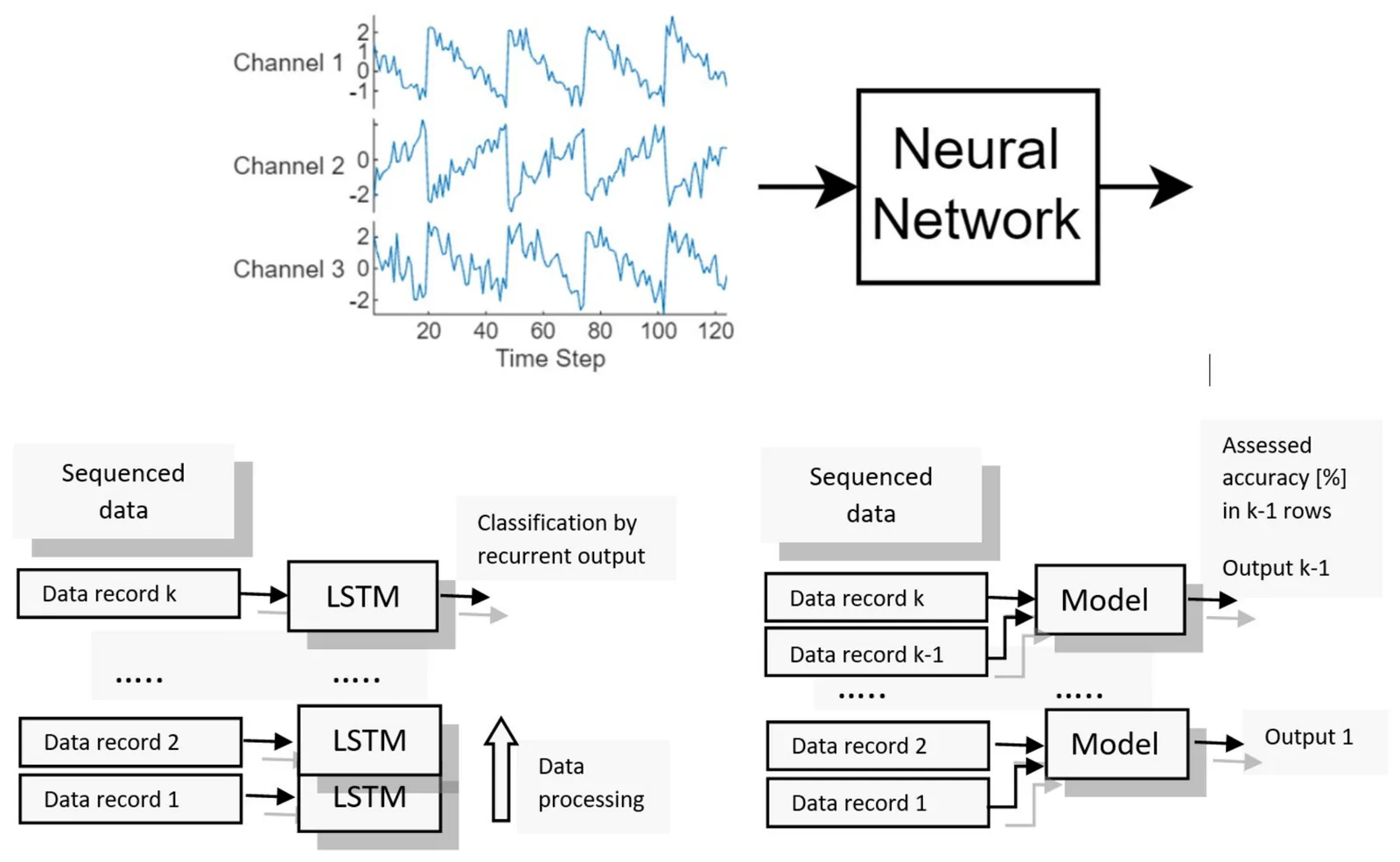
One-session sequence (recurrent or percentage output for all session rows) and sequence-to-sequence classification (separate output for a few lagged input–output data samples).

Two different key approaches in basic mindset identification have been compared and evaluated. The first sequence-to-one-output procedure processes all session row data in computing the percentage of true/false row-label responses or internal memory time–state gate-actualising cells, i.e., evaluating all session input with the same associated label all together (at the end time). The second sequence-to-sequence output approach evaluates each data record with a lagged input separately, i.e., classifies each single row with respect to previous time-delayed data so that it is statistically interpreted with overall classification accuracy at the session end (labelled with the same class):

1. Modular evolutionary DfL based on PDE node transform solutions was compared with MATLAB 2024a Deep Learning for Classification (DLC) using LSTM. The LSTM neural architecture is an advanced recurrent network (RN) type that can reveal and capture long-term dependencies in partial time-delayed steps on lagged data sequences. Standard RNs are limited in their ability to learn longer-term data relations in practice. To address this issue, LSTM networks include extra node gates to control the output information in the hidden cell propagated to the next hidden state, where *h_t_* is the output of a hidden state, and *c_t_* is the cell state at a time *t*. The complete sequence *y*_1_, …, *y_T_* or the last time output *y_T_*, equivalent to hidden states *h*_1_, …, *h_T_* or *h_T_*, is processed in an output classification layer (Figure 4). Cell gates enable effective learning of relevant long-term relationships in the data (forgot/update). A lower sensitivity to the time delay window improves the LSTM capability in sequential data analysis (D. Long Short-term Memory (LSTM) network https://www.mathworks.com/discovery/lstm.html?s_tid=srchtitle_support_results_2_LSTM. https://www.mathworks.com/help/deeplearning/ug/long-short-term-memory-networks.html) (E. MATLAB—Deep Learning neural network for Classification (DLC) https://www.mathworks.com/help/deeplearning/ug/deep-learning-in-matlab.html, www.mathworks.com/help/deeplearning/ug/classify-sequence-data-using-lstm-networks.html. Classify ECG Signals Using Long Short-Term Memory Networks https://www.mathworks.com/help/deeplearning/ug/classify-ecg-signals-using-long-short-term-memory-networks.html (all accessed on 18 September 2026)).
2. The DCN progressively evolves a binomial multilayer structure to produce node PDE components, which was compared with a variety of MATLAB classifiers in the implementation and evaluation of the second-sequence input–output computing methodology in the processing of time-lagged inputs. The Classification Learner App (CLA) toolbox allows exploring selected input features in specified validation tests to assess precision [%] in modelling experiments (H. DCN C++ parametric software with one merged Excel EEG data set: https://drive.google.com/drive/folders/1ZAw8KcvDEDM-i7ifVe_hDoS35nI64-Fh?usp=sharing. https://nextcloud.vsb.cz/s/EFDJ7mRyxfHkjSc). MATLAB ML methods perform automatic self-optimisation training to find the best model classifier, obtaining maximum accuracy in set tests (F. MATLAB—Classification Learner App (CLA) https://www.mathworks.com/help/stats/classificationlearner-app.html. Choose Classifier Options https://www.mathworks.com/help/stats/choose-a-classifier.html. Ensemble algorithms www.mathworks.com/help/stats/ensemble-algorithms.html). A shallow neural network (SNN) type of MATLAB was used in additional comparative experiments, but without obtaining acceptable results in recognition of self-labelled EEG data patterns (Figure 5) (G. Shallow Neural Network (SNN) https://www.mathworks.com/help/deeplearning/ref/network.train.html. Improve Shallow Neural Network Generalisation and Avoid Overfitting https://www.mathworks.com/help/stats/fitcnet.html (all accessed on 18 September 2026)).

## 5. AI Methods Applied in EEG Mental Classification Experiments

### 5.1. Differential Computing Network

The innovative Differential Computing Network (DCN) is an atypical free-form neuro-computing/maths fused modelling tool developed by the author. It combines the self-adaptive and evolutionary principles of ML and AI with the numerical procedures and definitions of OC. DBN parses the general definition of input *n* partial linear differential equations (PDEs) in a reduced sum set of two input converts of recognised orders in the transformation PDE nodes (1).

(1) $$A\frac{\partial^{2}u}{\partial x_{1}^{2}}+B\frac{\partial^{2}u}{\partial x_{1}x_{2}}+C\frac{\partial^{2}u}{\partial x_{2}^{2}}+D\frac{\partial u}{\partial x_{1}}+E\frac{\partial u}{\partial x_{2}}+Fu=G$$

(2) $$u=\sum_{k=1}^{\infty }u_{k}$$

A, B, C, …, G are functional parameters of the input variables x_1_, x_2_ for the unknown u function definition.

DfL allows modelling uncertain and chaotic dynamic systems whose behaviour is expressed with difficulty by an exact numerical formulation. The progressive evolution of DCN structures comprises selective feature extraction that mainly overcomes conventional regression or ML computing, which requires pre-processing data input. It gradually develops a multilayer binomial tree (BT), by adding node by node in the processing layer, to compose and select in each of them applicable PDE modules. The node PDE components are tested separately to add/extract the best one in/from the model sum. The progressive model expansion is based on Goedel’s incompleteness theorem in modular PDE decomposition to achieve training and testing optima. Each BT node selects applicable variables, produced by nodes in back-connected layers, to determine sub-PDE forms in employing ML-adapted procedures of OC. The modular DfL concept can represent complex unknown systems, described by dozens of periodic data variables with hidden correlation patterns (H. DCN C++ parametric software with one merged Excel EEG data set: https://drive.google.com/drive/folders/1ZAw8KcvDEDM-i7ifVe_hDoS35nI64-Fh?usp=sharing. https://nextcloud.vsb.cz/s/EFDJ7mRyxfHkjSc (accessed on 18 September 2026)). Several types of conversion functions in the OC definitions (polynomial, periodic, power) are available to relate *u* derivatives to an appropriate substitution form in the initial PDE (1) for selected node input couples:

1. **The polynomial transformation** of an ordinary differential equation (ODE) is based on an OC formulation for the Laplace L transform of the n^th^ derivative *f*(*t*) in relation to the initial states (3):

(3) $$L\left\{\left.f^{\left(n\right)}(t)\right\}\right.=p^{n}F(p)-\sum_{k=1}^{n}p^{n-i}f_{0+}^{\left(i-1\right)}L\left\{\left.f(t)\right\}\right.=F(p)$$

*f*(*t*), *f*’(*t*), …, *f*^(*n*)^(*t*)—*continual in* <0+, ∞>; *p*, *t*—*complex and time variables in the L*—*transform.*

The derivatives *f*(*t*) are converted by the OC definition (3) to *F*(*p*), which are L images of the original *f*(*t*) expressed by *p* in the complex linear equations. The *F*(*p*) is represented by a pure ratio and can become a summation form of the elementary root fractions (4).

(4) $$F(p)=\frac{P(p)}{Q(p)}=\frac{Bp+C}{p^{2}+ap+b}=\sum_{k=1}^{n}\frac{A_{k}}{p-\alpha_{k}}$$

*B*, *C*, *A_k_*—*fractional elements*; *a*,*b*—*polynom. Param*; *α*_1_, *α*_2_, …, *α_k_*—*simple Q*(*p*) *roots* (*real*).

Polynomial replacements for the derivatives *f*(*t*) (3) result in ratios related to the initial forms of ODE (1). The ratios correspond to the complex transformed *f*(*t*) that is to be restored inversely using the reverse definition of OC (5). The secondary inverse operation L of OC reconstructs an approximate of *f*(*t*) (5) to obtain the real function by solving its ODE (1).

(5) $$F(p)=\frac{P(p)}{Q(p)}=\sum_{k=1}^{n}\frac{P(\alpha_{k})}{Q_{k}(\alpha_{k})}\cdot \frac{1}{p-\alpha_{k}}f(t)=\sum_{k=1}^{n}\frac{P(\alpha_{k})}{Q_{k}(\alpha_{k})}e^{\alpha_{k}\cdot t}$$

*P*(*p*)*, Q*(*p*)—*s*-*1*, *s degree polynomials*; *α*_1_, *α*_2_, …, *α_n_*—*simple Q*(*p*) *roots* (*real*).

The inverse operation applied to the ratio *F*(*p*) of an analogously converted node PDE (6) is represented by the angle exponent of two inputs *φ = arctg*(*x*_2_*/x*_1_). Its real counterpart *u* is recovered from a transformation L node in this way to obtain the fractional *y_j_* PDE solution (1), which is demonstrated in Equation (6).

(6) $$y_{1}=w_{1}\frac{b_{0}+b_{1}x_{1}+b_{2}sig(x_{1}^{2})+b_{3}x_{2}+b_{4}sig(x_{2}^{2})}{a_{0}+a_{1}x_{1}+a_{2}x_{2}+a_{3}x_{1}x_{2}+a_{4}sig(x_{1}^{2})+a_{5}sig(x_{2}^{2})}\cdot e^{\phi }$$

*y_j_*—*partial sub-PDE solutions of node u_k_ functions*; *sig*—*sigmoidal transformation*.

*φ = arctg(x*_2_*/x*_1_*)*—*angle of 2-input variables x*_1_, *x*_2_; *a_i_*, *b_i_*, *w_j_*—*polynom. parameters and term weights*.

**Figure 6:**
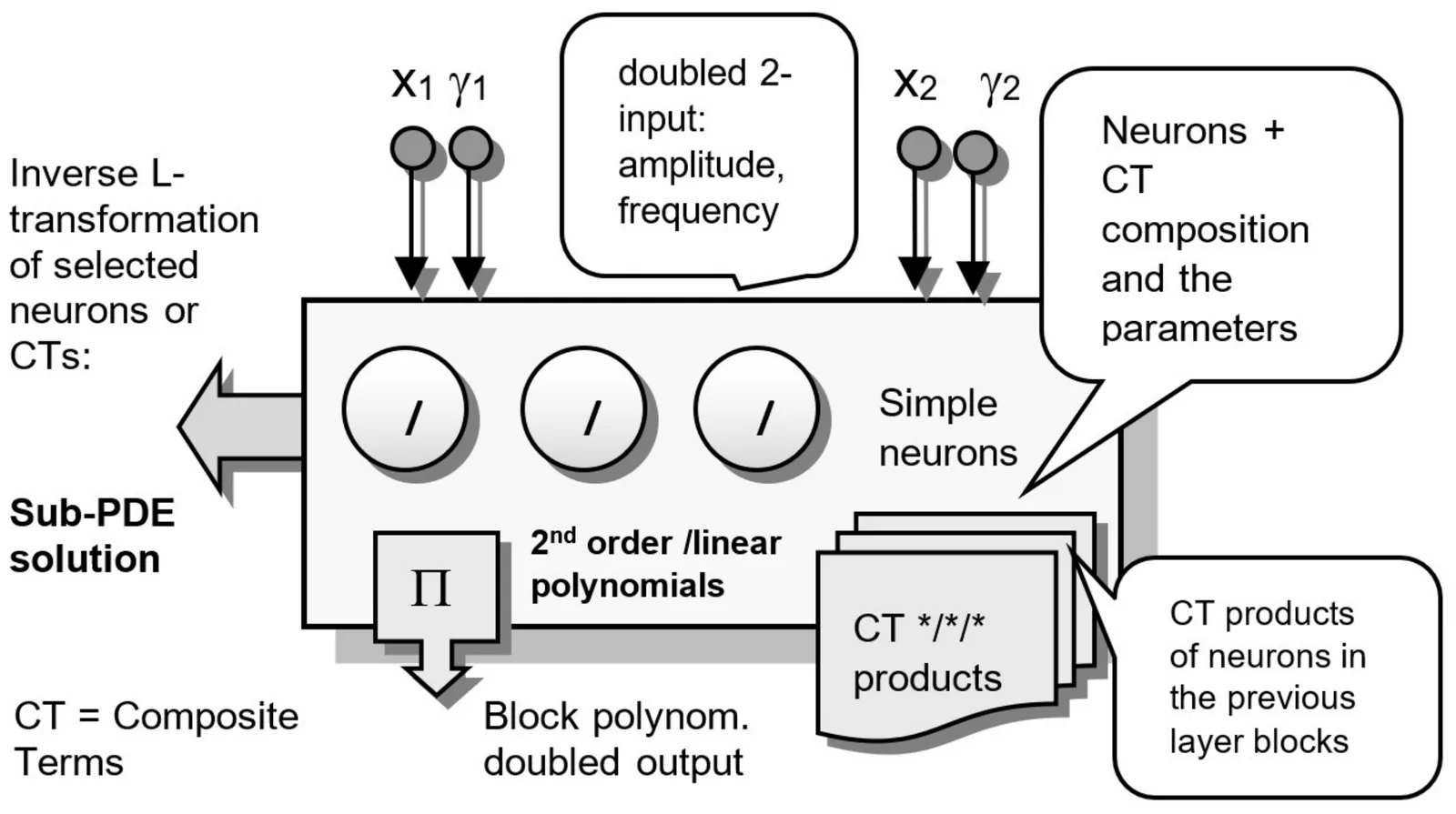
DCN blocks generate node sub-PDE transforms in composing the simple or composite model PDE-modules.

Appropriate first- or second-order multinomial members are examined in order to be selected in the numerator in composing two-variable ratio terms in nodes (6). The same complete second-order multinomial in the denominator of the applied PDE term is used to calculate the node *o_i_* output propagated to the next layer. Block co-produced terms (i.e., sum neurones in this context) and their polynomials are shared by all back-composed Composite Terms (CTs) in the products of input–output connected next-layer blocks (Figure 6).

2. **The periodic transformation** assumes that the real *f*(*t*) is a periodic function defined in the ODE. Its derivatives are converted to sine and cosine L equivalents to be restored again in the approximate original by a reverse L operation (7). This expanded definition is applied to real amplitudes *x*_1_, *x*_2_ and imaginary phases *γ*_1_*, γ*_2_ of the processing data in a doubled complex form to obtain convenient L representation of the sub-PDE transformers at nodes (8).

(7) $$\left(t)=\sum_{k=1}^{r}\frac{P(\alpha_{k})}{Q^{\prime }(\alpha_{k})}e^{\alpha_{k}\cdot t}+2\sum_{k=1}^{s}e^{\beta_{k}\cdot t}(a_{k}\cdot \cos\gamma_{k}t-b_{k}\cdot \sin\gamma_{k}t\right)$$

The periodic conversion of node PDE for the doubled input *x*_1_*, x*_2_ and *γ*_1_*, γ*_2_ is demonstrated in Equation (8):

*y_i_* = [*a*_1_*x*_1_·*cos*(*b*_1_2*π*·*γ*_1_·*t* + *b*_0_) − *a*_2_*x*_2_·*sin*(*b*_2_2*π*·*γ*_2_·*t* + *b*_0_)]·*e^ϕ^* (8)

*y_i_*—*periodical output of a node PDE-term*; *γ*_1_*, γ*_2_*—phases [radian]*; *x*_1_*, x*_2_*—real amplitudes*, ***a***, ***b****—coeff.*

In the case of time periods in the data series, a Fourier representation in the corresponding PDE conversion (8) can be used considering the time *t derivative* (9).

*Y_i_* = [*a*_1_*x*_1_·*cos*(*b*_1_*π·t* + *b*_0_) + *a*_2_*x*_2_*·sin*(*b*_2_*π·t* + *b*_0_)] · *e^ϕ^* (9)

*y_i_—Fourier representation of a node PDE-term for t—time; x*_1_, *x*_2_*—amplitude variables; φ* = *γ*_1_ − *γ*_2_.

The wave *p_i_* output is computed in the block nodes analogously to the *o_i_* amplitude polynomial (6) as a simple weighted linear sum to be processed in the doubled form in the next layers:

*p_i_* = *b*_0_ + *b*_1_*γ*_1_· + *b*_2_*γ*_2_ (10)

The processed data can be expressed in the complex form of the Euler conjugate *c* (11) to relate the OC *F*(*p*) transform to the real original *f*(*t*) (5). The radius *r* (amplitude) can be considered a reference to the rational part, while the phase parameter *φ* = *arctg*(*x*_2_*/x*_1_*)* corresponds to the inverse L-recovery for *F*(*p*) (5).

*C* = *Re + Im* = *x*_1_ + *i·x*_2_ = *r·e^i·^^φ^ = r·*(*cosφ + i·sinφ*) (11)

3. **Power conversion** uses simple linear and power functions, resulting from the rational definition of terms (6), to allow the representation of simple linear or parabolic data relations by a sum PDE solution (1). No exponential inverse L-transformation of these polynomial elements is necessary (12), as they do not result from the PDE conversion for the L derivatives. These components are inserted into the PDE-based model to refine its sum output.

*y_i_* = *a*_0_ + *a*_1_*x*_1_      *y_i_* = *a*_0_ + *a*_1_*x*_1_ + *a*_2_*x*_2_ + *a*_3_*x*_1_*x*_2_          *y_i_* = *a*_0_ + *a*_1_*x*_1_ + *a*_2_*x*_1_^2^ (12)

All blocks produce simple rational, periodic, or power terms, that is, sum neurones in the selected BT nodes (Figure 6). CTs can be extra produced, except for the first layer, to capture hidden interrelationships between multilayer variables of internal and external functions. CTs are back-composed as chain products from neurones built in back-connected blocks in the tree structure, employing the composite function derivation rules, as generalised in Equation. The final inverse exponential transform is applied to a product term in the starting block to obtain the original defined by its composite node PDE of a CT [25].

**Figure 7:**
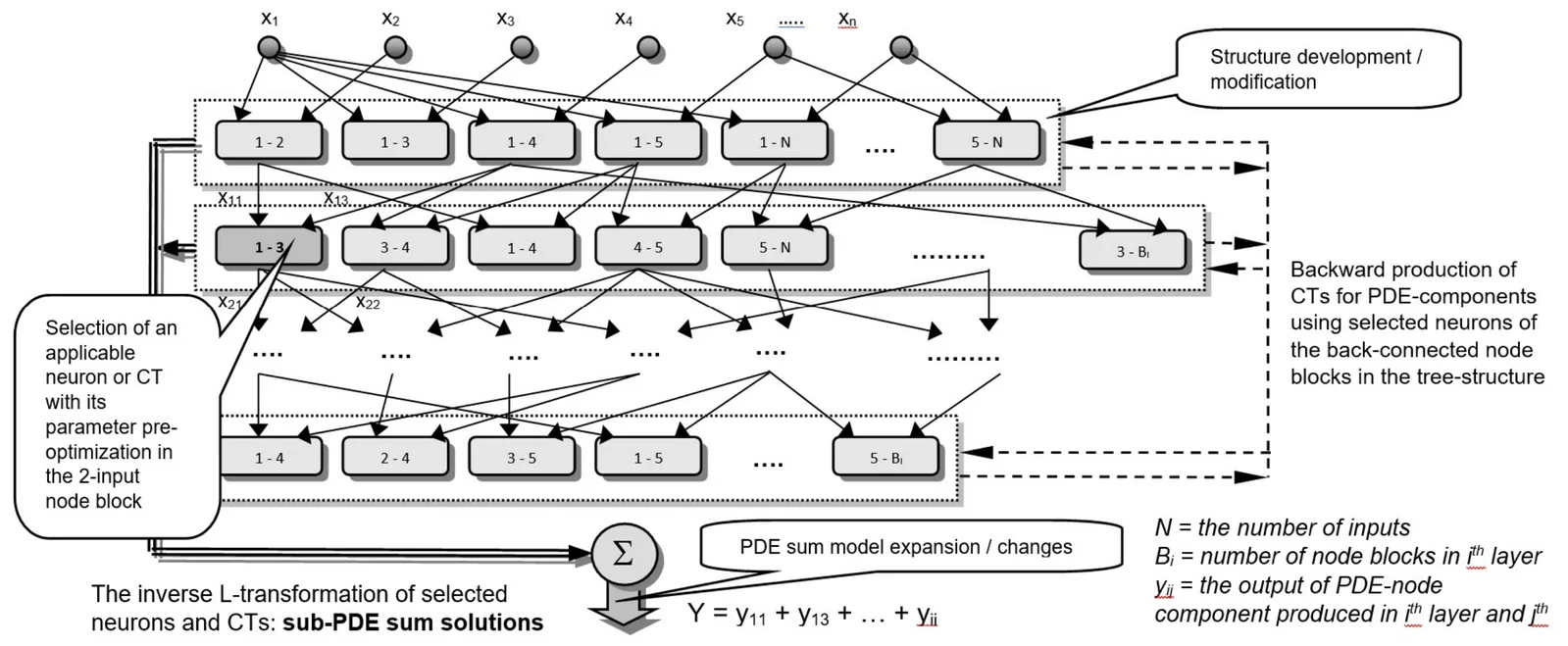
DCN selects the most valuable two input nodes in an evolved binomial tree structure to form backward products for optimal PDE component solutions summed in the model output.

Optimisation algorithms add or skip node by node in evolving a BT structure to choose or validate each specific *F*(*p*) conversion type for the *x*_1_, *x*_2_ node PDE terms to better approximate the desired target. The sum of two variable originals *u_k_* of selected BT nodes (Figure 7) represents a final combination PDE model of the n-variable *u* summation function (13).

(13) $$Y=\frac{1}{k}\sum_{i=1}^{k}y_{i}\;\;\;\;\;\;\;\;k=\mathrm{the}\;\mathrm{number}\;\mathrm{of}\;\mathrm{active}\;\mathrm{neuron}\;\mathrm{or}\;\mathrm{CT}\;\mathrm{node}\;\mathrm{PDE}\;\mathrm{sum}\;\mathrm{terms}$$

DCN iteratively searches for the best inputs *x_i_*, *x_j_* in each actualised node by forming and examining its standalone PDE components to insert a new or change an existing one in the layer (Figure 7). The assessed input combination couples used by the minimum error terms in a layer verification node are gradually applied to evaluate all sub-PDE components (to better approximate the desired data). The best node 2-inputs are ranked in each layer separately, not including their components in the overall model first, which essentially speeds up computing and searching in the exponential combinatorial space. After the initial input ranking, the algorithm processes the best listed node elements to try to include their PDE terms, one by one, in the output model [26]. The DCN gradually expands its sum model by inserting selected block PDE solutions of neurones (6) or CT (14), produced at the detected nodes. This step-by-step model expansion, using optimal node PDE components, is based on the Goedel incompleteness theorem [27]. Tree-embedded nodes are not removed from the BT in the next iterations, as they produce output processed in the next layer. The DCN dynamically evolves or modifies its BT structure in each training cycle. The nodes and layers are gradually added to a BT by expanding the bound PDE model with the best PDE terms until improvements in the approximate sum output are feasible [28]. Some node blocks can be inactivated to eliminate redundant components (using the same multilayer composite variables) in optimising backward DCN structures. Parameter updates, resulting from the error derivatives of solution functions (term derivatives), are backpropagated with respect to the output errors, using the negative gradient [29].

### 5.2. Deep Learning Using the LSTM Network

The key component of an LSTM-based DL classifier is the input sequence layer, which feeds sequenced series into the LSTM layer learning long-term iterative data relations between time-delayed inputs (E. MATLAB—Deep Learning neural network for Classification (DLC) https://www.mathworks.com/help/deeplearning/ug/deep-learning-in-matlab.html, www.mathworks.com/help/deeplearning/ug/classify-sequence-data-using-lstm-networks.html. Classify ECG Signals Using Long Short-Term Memory Networks https://www.mathworks.com/help/deeplearning/ug/classify-ecg-signals-using-long-short-term-memory-networks.html (all accessed on 18 September 2026)).

**Figure 8:**
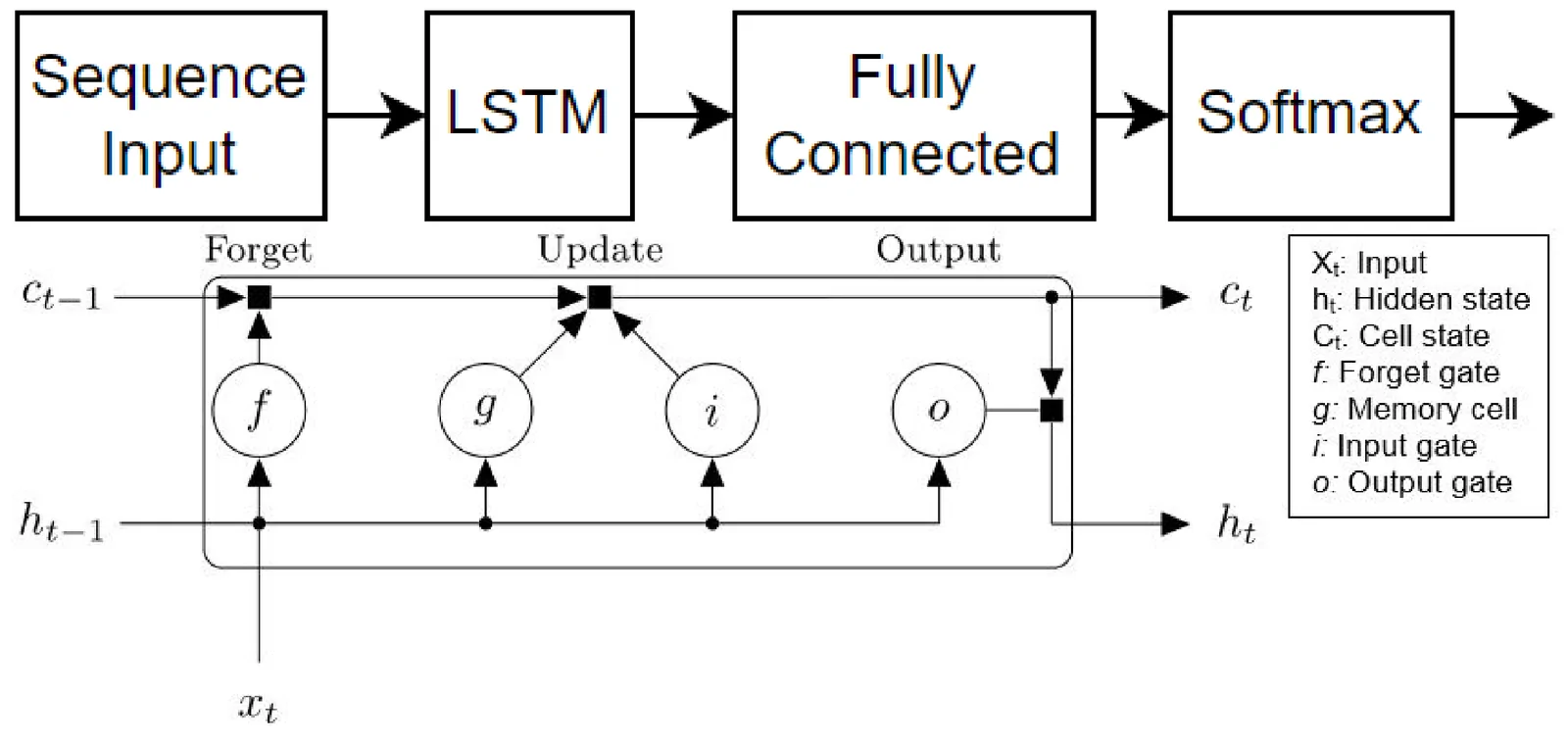
Gate input–output time flow in an LSTM layer (Figure 4) and the basic architecture of an LSTM neural network for DL classification (D. Long Short-term Memory (LSTM) network https://www.mathworks.com/discovery/lstm.html?s_tid=srchtitle_support_results_2_LSTM. https://www.mathworks.com/help/deeplearning/ug/long-short-term-memory-networks.html (accessed on 18 September 2026)).

LSTM states are defined by their hidden (output) and cell (actual) status. The hidden state at time step *t* contains the output *h_t_*. of information learnt from the previous time steps. At each time step *t*, cells add or remove information to/from the previous state by controlling their updates with gate weights (Figure 8). The current cell/hidden state (*c_t_*_−1_, *h_t_*_−1_) input is used to compute the output *h_t_* and the updated cell state *c_t_* at the next time *t* of the sequence. The gate activation functions are by default hyperbolic tangent and sigmoid. The adaptable gate weights in an LSTM layer are input, recurrent, and bias (D. Long Short-term Memory (LSTM) network https://www.mathworks.com/discovery/lstm.html?s_tid=srchtitle_support_results_2_LSTM. https://www.mathworks.com/help/deeplearning/ug/long-short-term-memory-networks.html (accessed on 18 September 2026)).

The forget gate (14) decides which information to keep and which to forget, applying sigmoid activation to the weights *W_f_* multiplied by the concatenated previous hidden state *H_t_*_−1_ and the current input *X_t_*, plus the bias *b_f_*.

*F_t_* = *σ*(*W_f_ Z_t_* + *b_f_*) (14)

The candidate gate/memory (15) processes new information *Z_t_* (=*H_t_*_−1_·*X_t_*) shown to the LSTM layer multiplied with weights and biases W_c_ and *bc* by hyperbolic tangent activation.

*C_t_* = *tanh*(*W_c_ Z_t_* + *b_c_*) (15)

The input gate (16), analogously to (9), decides which information supplied to the network is remembered in the opposite of the memory state.

*I_t_* = *σ*(*W_i_ Z_t_* + *b_i_*) (16)

The output gate (17) again determines what information to remember between the 2-hidden states by the sum of bias *b_o_* and the product of the weights *W_o_* and the concatenated input *Zt*.

*O_t_* = *σ*(*W_o_ Z_t_* + *b_o_*) (17)

The final gate (18) usually does not regularly contain an activation function applied to the *H_t_* output, although it may be useful in some applications.

*Y_t_* = *W_y_ H S_t_* + *b_y_* (18)

DLT employs the basic LSTM architecture that can learn complex correlations from input–output samples. This hybrid type of recurrent network integrates multiple processing (in simple computing elements) with biologically inspired operation based on connectivity-adaptable gates. DLT networks require an experimental hand design for a defined replicate structure, which may also comprise different types of layers [30]. LSTM retains a hidden state *H_t_* with an extra memory cell state *C_t_*. Cell memory allows retaining information from long-term patterns, while hidden states usually hold short-term relationships learnt from data (Figure 7).

### 5.3. MATLAB Classification Learner App (CLA)

The CLA provides a variety of classification tools in supervised ML of automated training. It allows exploring data, selecting features, specifying validation schemes in learning, and final assessing results. The CLA toolbox includes the following classifiers: decision trees, ensembles, discriminant analysis, SVM, linear and logistic regression, nearest neighbours, naive Bayesian techniques, kernel approximation, and neural nets.

**Figure 9:**
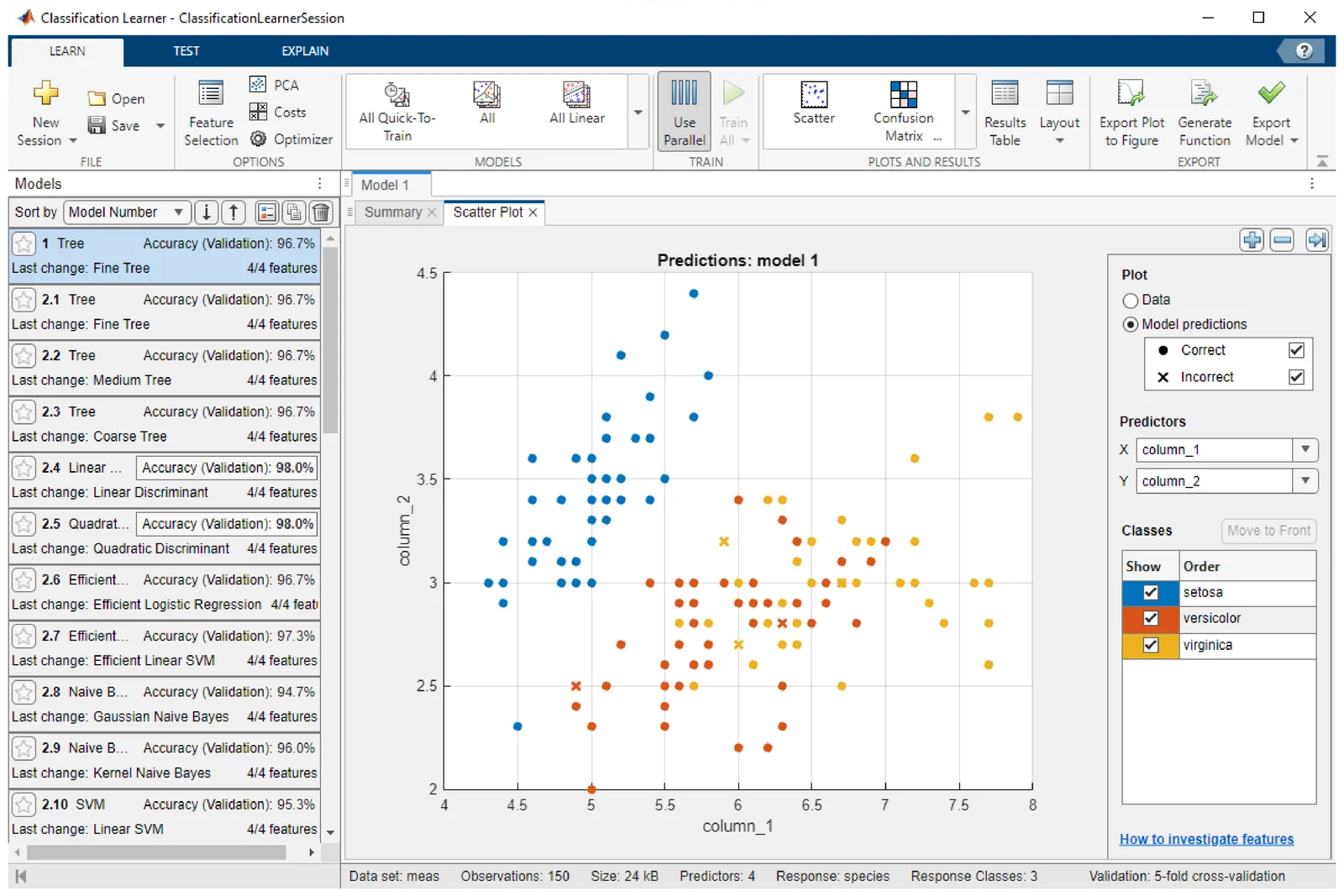
Graphical visualisation of a tree model validation (ranking) in the Classification Learner App (CLA) using the MATLAB scatter plot (F. MATLAB—Classification Learner App (CLA) www.mathworks.com/help/stats/classificationlearner-app.html. Choose Classifier Options www.mathworks.com/help/stats/choose-a-classifier.html. Ensemble algorithms www.mathworks.com/help/stats/ensemble-algorithms.html).

A known set of input-data observations with the related desired responses (class labels) are used to train and evaluate CLA models that generate prediction responses for new data input, as is routine in ML CLA searches, and assess the best classification model type by the test error minima in validation (Figure 9). The final classifier is exported to the MATLAB workspace (or its code is generated to recreate it) to be applied to a new input in the prediction (F. MATLAB—Classification Learner App (CLA) www.mathworks.com/help/stats/classificationlearner-app.html. Choose Classifier Options https://www.mathworks.com/help/stats/choose-a-classifier.html. Ensemble algorithms https://www.mathworks.com/help/stats/ensemble-algorithms.html (accessed on 18 September 2026)).

### 5.4. Shallow Neural Network (SNN)

**Figure 10:**
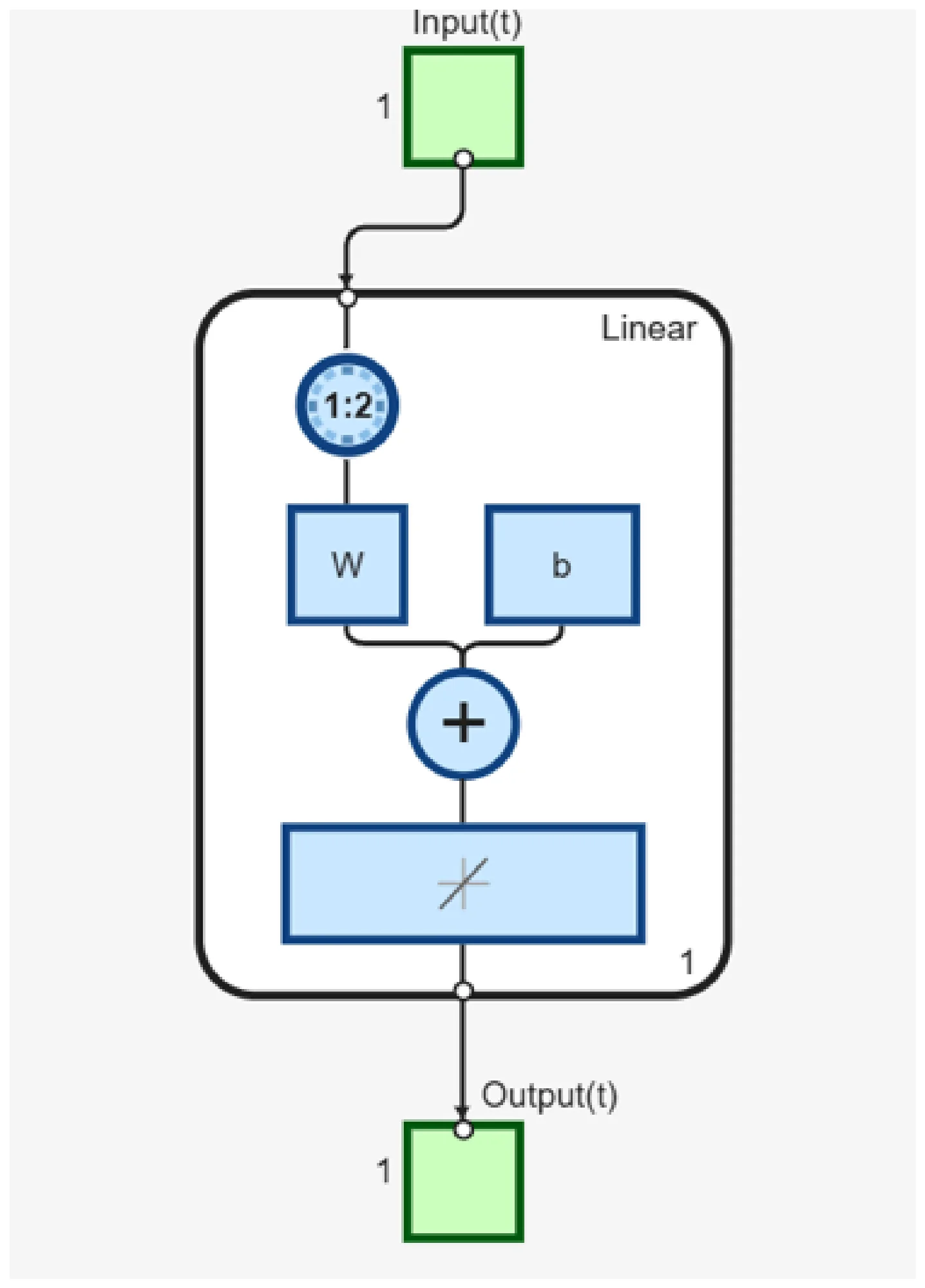
A MATLAB designer with shallow neural network (SNN) for pattern recognition (G. Shallow Neural Network (SNN) https://www.mathworks.com/help/deeplearning/ref/network.train.html. Improve Shallow Neural Network Generalisation and Avoid Overfitting https://www.mathworks.com/help/stats/fitcnet.html (accessed on 18 September 2026)).

The applied SNN is a two-layer feedforward NN with a sigmoidal transfer function in the hidden layer and a softmax transfer in the output layer. The size of the hidden layer corresponds to the number of hidden neurones (by default 10). The MATLAB Network pane can be used to design the network architecture. The number of output neurones is set to 2, which corresponds to the number of classes in the response data (Figure 10).

Each SNN training can result in different model solutions determined by random initial weights and biases influenced also by various data divisions into training, validation, and testing sets. For these reasons, an SNN trained on the same problem can produce different outputs for the same input. To ensure better classification accuracy, it should be re-trained several times. A larger training set or an increased number of inputs (if available) can be used with another algorithm in learning. Recent techniques have been developed to improve initial solutions by increasing the generalisability of SNNs and avoiding overfitting (G. Shallow Neural Network (SNN) https://www.mathworks.com/help/deeplearning/ref/network.train.html. Improve Shallow Neural Network Generalisation and Avoid Overfitting https://www.mathworks.com/help/stats/fitcnet.html (accessed on 18 September 2026)).

## 6. EEG Experiments in Recognising 2-Primary Mental States

Concentration levels comprising explicit score values in the interval <0, 1> from the original data set (C. Personal concentration data sets (80 .CSV files) https://www.kaggle.com/datasets/dqmonn/personal-eeg-tasks) were redistributed into two main relaxation and concentration categories, corresponding to mental states 0 and 1 (A. Muse 3-channel EEG headset https://choosemuse.com/pages/science (all accessed on 18 September 2026)). The simple home Muse 2 front headband is supposed to not allow for detailed monitoring and discriminability in the measured EEG wave characteristics. Therefore, the initial self-concentration score assessed by real values was reduced only to the class labels ‘0’ for values below 0.5 and ‘1’ related to all values equal to or above the level of 0.5. The two relabelled elementary mental states were experimentally identified using the two key methodologies applying various recent classification techniques described above (Section 4 and Section 5). The complete data set consisting of 80 separate files, each with a self-labelled EEG session, was divided into two training/validation and prediction sets of 50–50% and 2/3-1/3 of the merged data file in the following modelling experiments. The identification fruitfulness of the models is calculated as the percentage ratio of the true response of the model *y_T_* to the total number of labels assigned *k* rows/sessions (19):

*Accuracy* = 100 · *y_T_*/*k* [%] (19)

**Figure 11:**
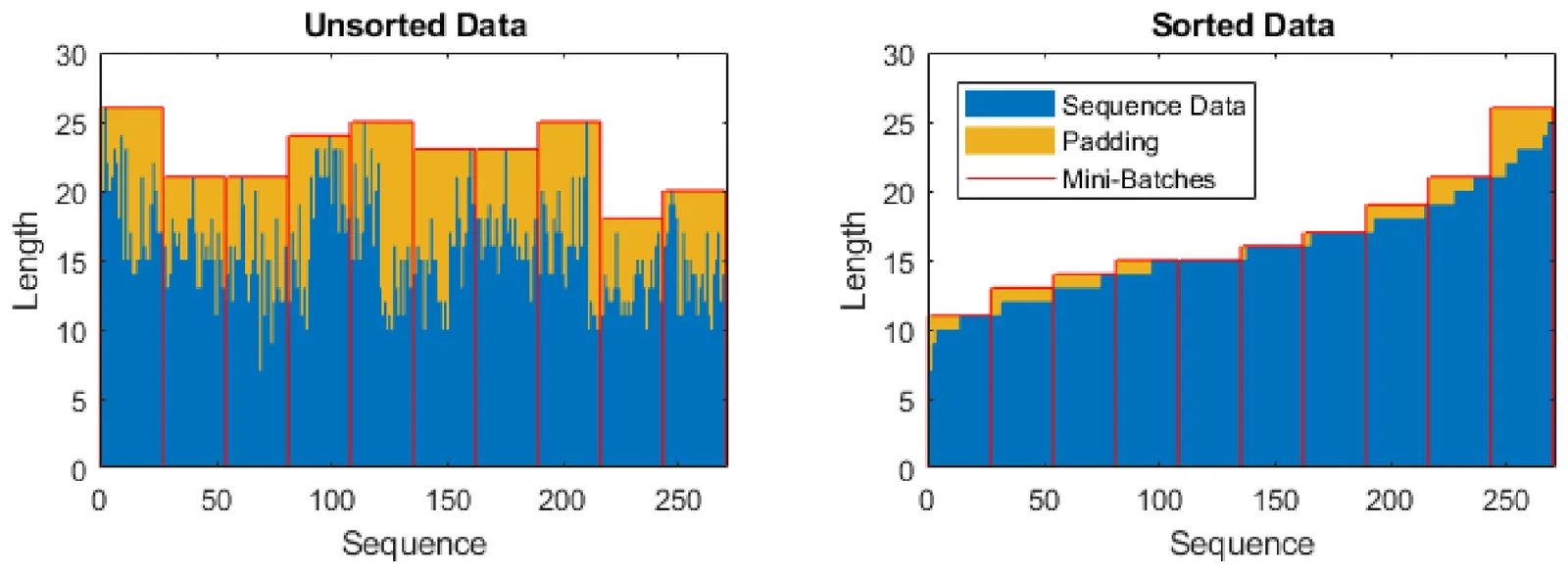
The effect of DLC sequence padding before and after data sorting in MATLAB (E. MATLAB—Deep Learning neural network for Classification (DLC) https://www.mathworks.com/help/deeplearning/ug/deep-learning-in-matlab.html, www.mathworks.com/help/deeplearning/ug/classify-sequence-data-using-lstm-networks.html. Classify ECG Signals Using Long Short-Term Memory Networks https://www.mathworks.com/help/deeplearning/ug/classify-ecg-signals-using-long-short-term-memory-networks.html (all accessed on 18 September 2026)).

DLC splits the training data into mini-batches and pads the sequences to be of the same length. To avoid higher paddings, which does not positively impact the performance of the LSTM network, the training data can be sorted by their part lengths with a chosen mini-batch size into sequences having a similar size (Figure 11) (E. MATLAB—Deep Learning neural network for Classification (DLC) https://www.mathworks.com/help/deeplearning/ug/deep-learning-in-matlab.html, www.mathworks.com/help/deeplearning/ug/classify-sequence-data-using-lstm-networks.html. Classify ECG Signals Using Long Short-Term Memory Networks https://www.mathworks.com/help/deeplearning/ug/classify-ecg-signals-using-long-short-term-memory-networks.html (all accessed on 18 September 2026)). Although training data were organised in the recommended form, this initial pre-processing mostly does not yield better prediction results on the untrained session sequences (sorted naturally in the same manner). The size of the input layer is equivalent to the number of EEG channels in the data recording (that is, 20 variables without time stamp). LSTM networks allow learning long-term dependencies between time steps in sequenced data. DLC used a bidirectional LSTM layer with 120 hidden units to compute the output for the last element in the time sequence. The bidirectional LSTM layer looks at time sequences in the data in both forward and backward directions. This type of layer learns from the full sequence of data at each time step, so it is used if the complete series at prediction times is known (as EEG records). In the case of forecasting an output for unavailable data input in the next time steps, it is obviously not applicable. A fully connected layer with a size that matches the number of classes (i.e., 2) supplies input to a MATLAB softmax layer (Figure 8).

**Figure 12:**
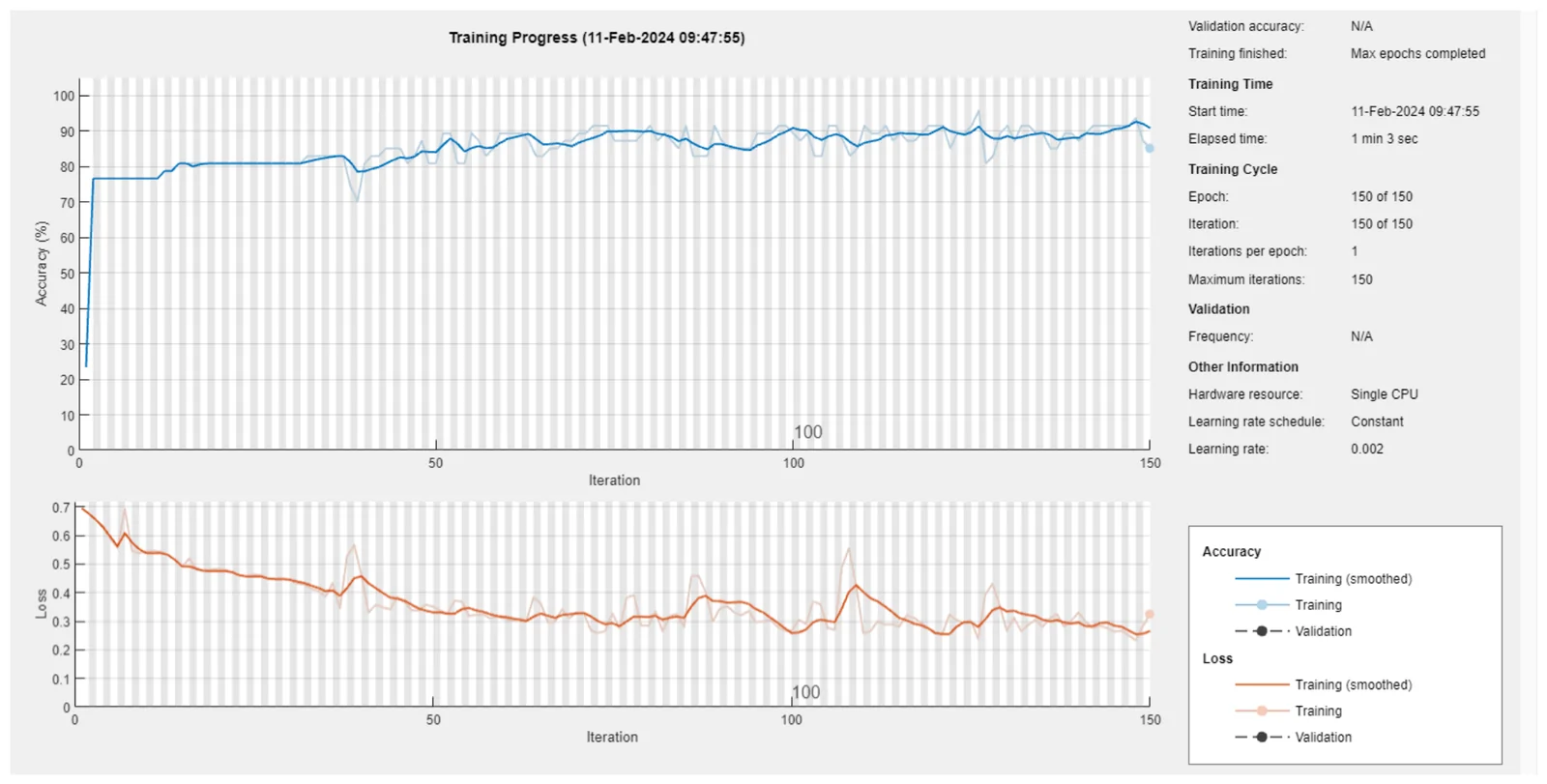
DLC training and validation in MATLAB using 2/3-1/3 data in training–prediction, without data sorting (E. MATLAB—Deep Learning neural network for Classification (DLC) https://www.mathworks.com/help/deeplearning/ug/deep-learning-in-matlab.html, www.mathworks.com/help/deeplearning/ug/classify-sequence-data-using-lstm-networks.html. Classify ECG Signals Using Long Short-Term Memory Networks https://www.mathworks.com/help/deeplearning/ug/classify-ecg-signals-using-long-short-term-memory-networks.html (all accessed on 18 September 2026)).

The training set contained 90% of the data, while the remaining 10% was reserved for model validation tests. DLC training was limited to 150 epochs, with the learning rate set at 0.002 and a gradient threshold of 1.0. The training loss is the cross-entropy decrease in each mini-batch, and with successful progress, it is minimalised. DLC models without session padding casually show unstable behaviour in training, with higher uncertainty (lower robustness) in processing new data in predictions, despite their high prediction accuracy. Processing a longer sequence of data to actualise the memory states in each next time-delay step results in higher variability in iteratively computed cell outputs and parameter updates. This phenomenon can be observed from the alternate progress of training loss without data sorting (Figure 12). Although mainly resulting in higher prediction accuracy, some model adaptation can fail, showing lower output precision when processing new data.

**Figure 13:**
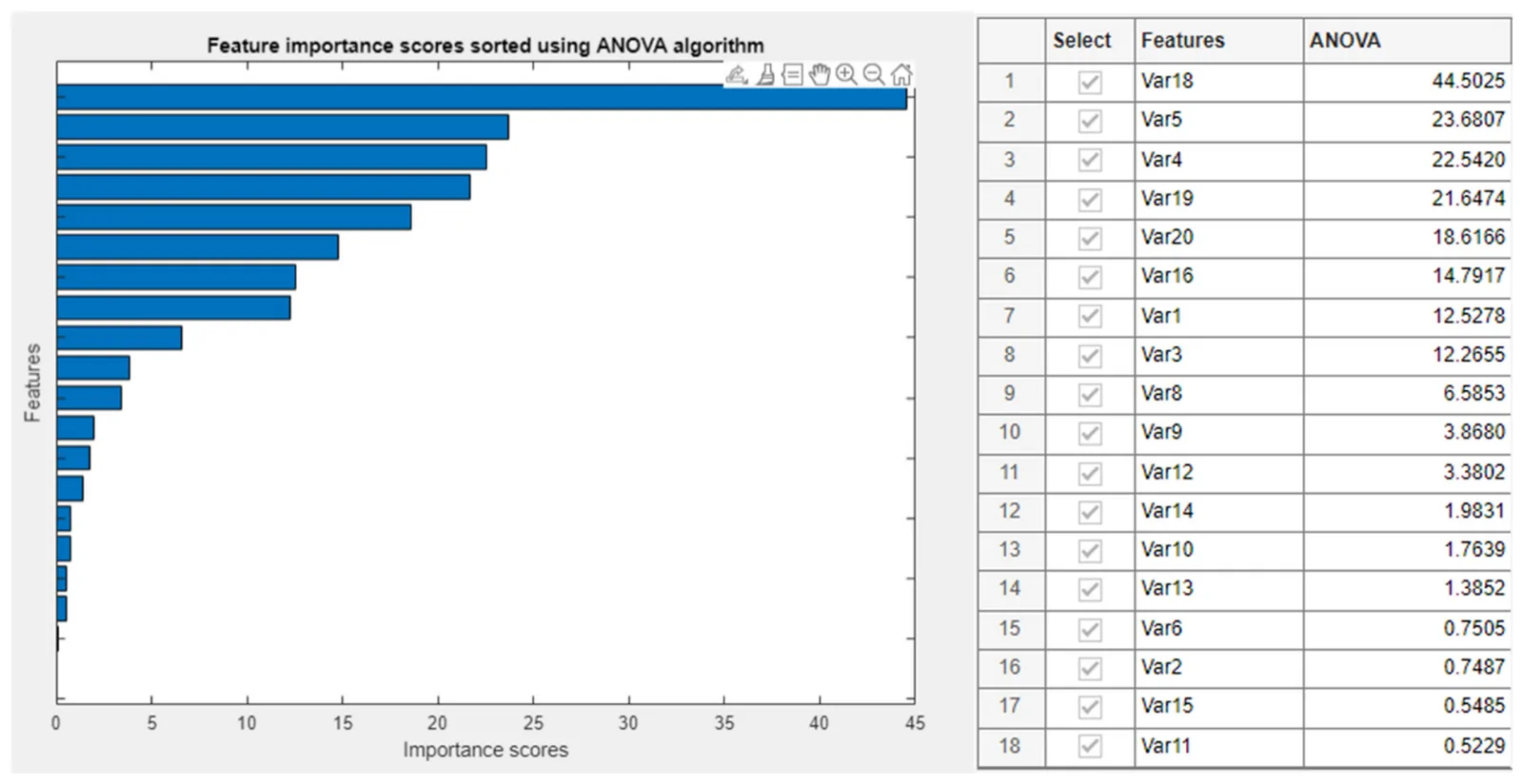
MATLAB feature ranking in CLA (F. MATLAB—Classification Learner App (CLA) https://www.mathworks.com/help/stats/classificationlearner-app.html. Choose Classifier Options https://www.mathworks.com/help/stats/choose-a-classifier.html. Ensemble algorithms https://www.mathworks.com/help/stats/ensemble-algorithms.html (all accessed on 18 September 2026)).

DCN, CLA, and SNN were compared in the second sequence-to-sequence multiple-output methodology using training and tests carried out in the same way, as described above, by DLC. DCN composed its models from selected PDE components produced in node-by-node-evolved BT structures (as presented in detail in Section 5.1). Each input amplitude is doubled by a mean frequency value from the corresponding five-band range (alpha, beta, gamma, delta, and theta) to be applied in an attempt at the overall model extension with a form of periodic sub-PDE component in an actualised BT node. Both the 2-input node amplitude and frequency data are used to compute the doubled multinomial (non-)linear node output. This complex input extension in DCN internal data processing seems to contribute to a better representation of EEG patterns in periodic node-defined PDE solutions [31]. DCN models were found to be the most successful at applying both the conventional row-by-row data sequence and whole session identification strategies [26]. CLA performs automated (possibly choosing classifier options) self-optimising training on a variety of ML techniques in searching and graphically comparing the best prediction models by assessing the test error minima (Figure 9). It offers a set of feature ranking algorithms (e.g., MRMR, Chi2, ReliefF, Anova, etc.) that can help reduce the size of the input vector by displaying a plot of importance scores for the sorted features (Figure 13). Principal component analysis (PCA) is also a built-in tool of CLA, reducing the dimensionality of the predictor space to prevent overfitting. PCA linearly transforms the predictors by removing redundant dimensions to generate a new set of input variables (F. MATLAB—Classification Learner App (CLA) www.mathworks.com/help/stats/classificationlearner-app.html. Choose Classifier Options https://www.mathworks.com/help/stats/choose-a-classifier.html. Ensemble algorithms https://www.mathworks.com/help/stats/ensemble-algorithms.html (all accessed on 18 September 2026)). PCA keeps the input components that explain 95% of the variance by default. However, the use of these feature selection algorithms did not improve CLA modelling, perhaps due to the lower dimension of the applied EEG data (20 amplitude inputs in all).

**Figure 14:**
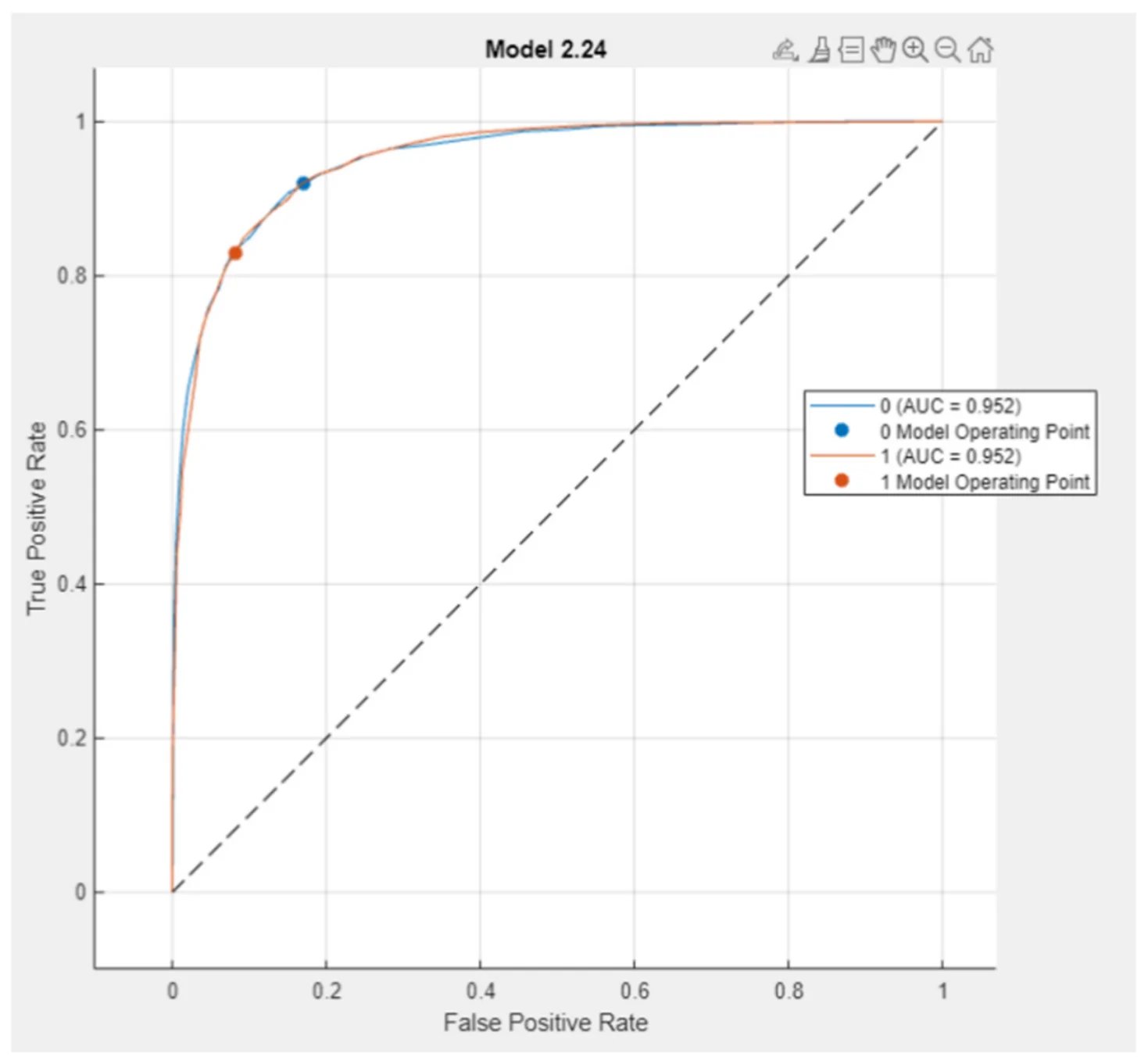
True–false positive CLA rates of the best EBT model (validation accuracy 88.5%) in the ROC validation curve, (classes 1, 0) 50–50% of the data used in the training–prediction (F. MATLAB—Classification Learner App (CLA) https://www.mathworks.com/help/stats/classificationlearner-app.html. Choose Classifier Options https://www.mathworks.com/help/stats/choose-a-classifier.html. Ensemble algorithms https://www.mathworks.com/help/stats/ensemble-algorithms.html (all accessed on 18 September 2026)).

In the testing, standard 5-fold cross-validation was used, analogously to the evolution of the DCN models. It performs multiple tests but protects the model from overfitting efficiently in small sets. This type of model validation allows training with all data and usually improves predictive accuracy. Finally, the best classification model is exported to the MATLAB workspace to make predictions for new data (F. MATLAB—Classification Learner App (CLA) https://www.mathworks.com/help/stats/classificationlearner-app.html. Choose Classifier Options https://www.mathworks.com/help/stats/choose-a-classifier.html. Ensemble algorithms https://www.mathworks.com/help/stats/ensemble-algorithms.html (all accessed on 18 September 2026)). The most successful CLA model was the Ensemble Bagged Tree (EBT), whose formation was limited by the maximal number of learners (iterations): 30 and 4826 splits using Bayesian optimisation. The performance of a classification ensemble is usually explored by plotting the receiver operating characteristic (ROC) (Figure 14).

Bagging (bootstrap aggregation) ensembles bag weak learners (decision trees) on a data set by generating various bootstrap replicas on the original data to grow decision trees on the subsets. Observation replacements are randomly selected in the data set. The tree members in the ensemble randomly select predictors for each decision split in a random forest to improve the accuracy of the EBT. By default, the number of predictors in the selection of a random split is equal to the square root of the number of predictors in the classification. Ensembles could be trained with only a few trees on observations that are in a bag of all trees to return the most probable class in a response. The EBT model finally averages the predictions over individual tree members for the new data input (F. MATLAB—Classification Learner App (CLA) https://www.mathworks.com/help/stats/classificationlearner-app.html. Choose Classifier Options https://www.mathworks.com/help/stats/choose-a-classifier.html. Ensemble algorithms https://www.mathworks.com/help/stats/ensemble-algorithms.html (all accessed on 18 September 2026)).

**Figure 15:**
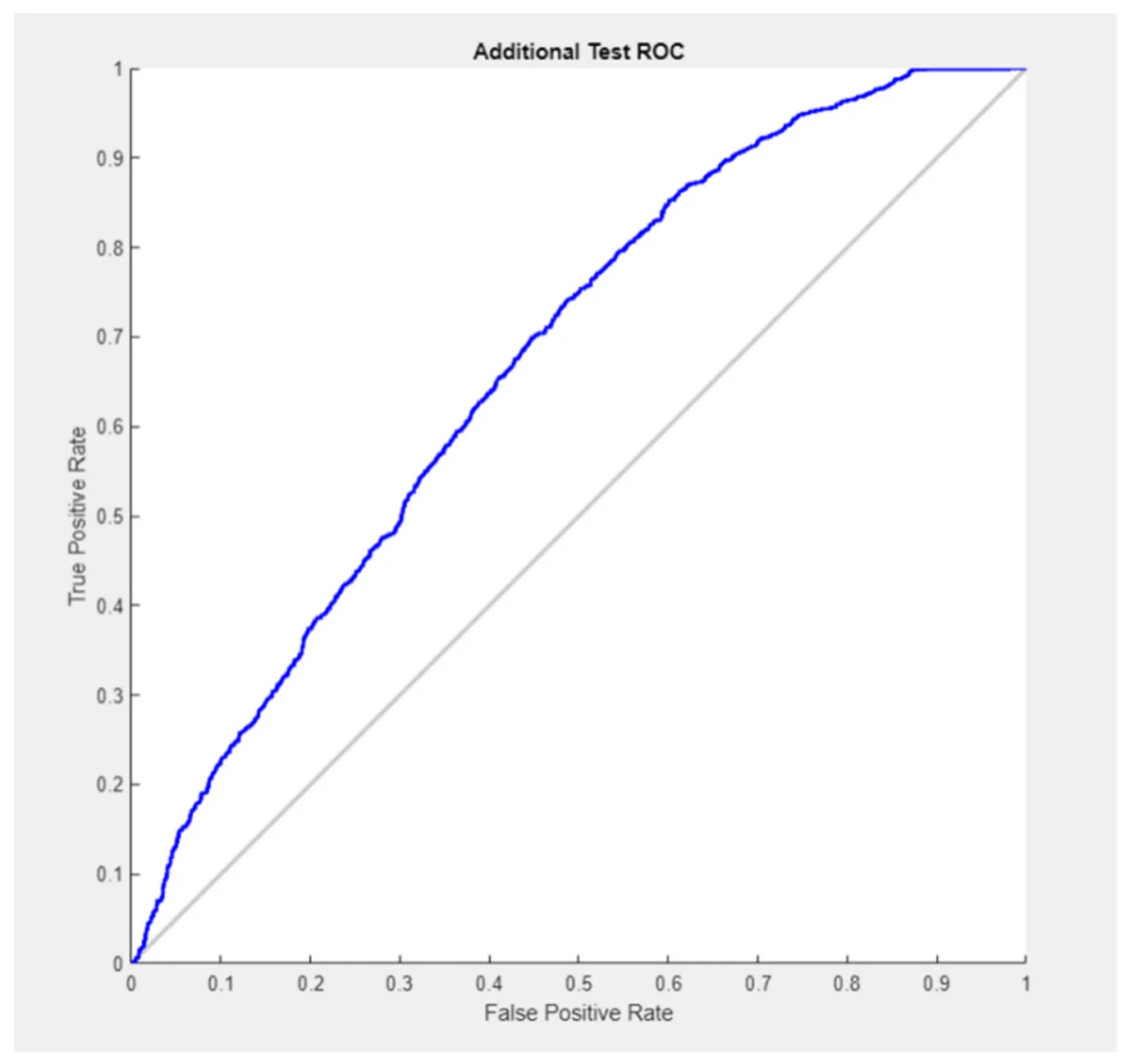
True–false positive rates of the SNN model (cross-entropy/error: training = 0.586/0.345 validation = 0.589/0.330) in the ROC validation curve (2/3-1/3 of the data used in training–prediction) (G. Shallow Neural Network (SNN) https://www.mathworks.com/help/deeplearning/ref/network.train.html. Improve Shallow Neural Network Generalisation and Avoid Overfitting https://www.mathworks.com/help/stats/fitcnet.html (all accessed on 18 September 2026)).

Although SNN models obtained low predictive accuracy, their model generalisation and results could be partially improved by using recommended training and data validation procedures or additional published strategies (Figure 15) (G. Shallow Neural Network (SNN) https://www.mathworks.com/help/deeplearning/ref/network.train.html. Improve Shallow Neural Network Generalisation and Avoid Overfitting https://www.mathworks.com/help/stats/fitcnet.html (all accessed on 18 September 2026)). However, all attempts to improve the SNN modelling did not yield better classification results (e.g., network architecture, hidden layer size, learning parameters, adaptation method, etc.).

## 7. Analytical Results and Evaluation of Experiments in the Identification of Key Mind Concentration/Relaxation States

The binary confusion matrix relates the positive ‘1’ and negative ‘0’ classes in the true and false model responses (Table 4) in the model evaluation (training/test validation or prediction).

Table 5 compares the DCN confusion results in predictive true/false model responses in the second row-to-row sequence evaluation methodology.

**Figure 16:**
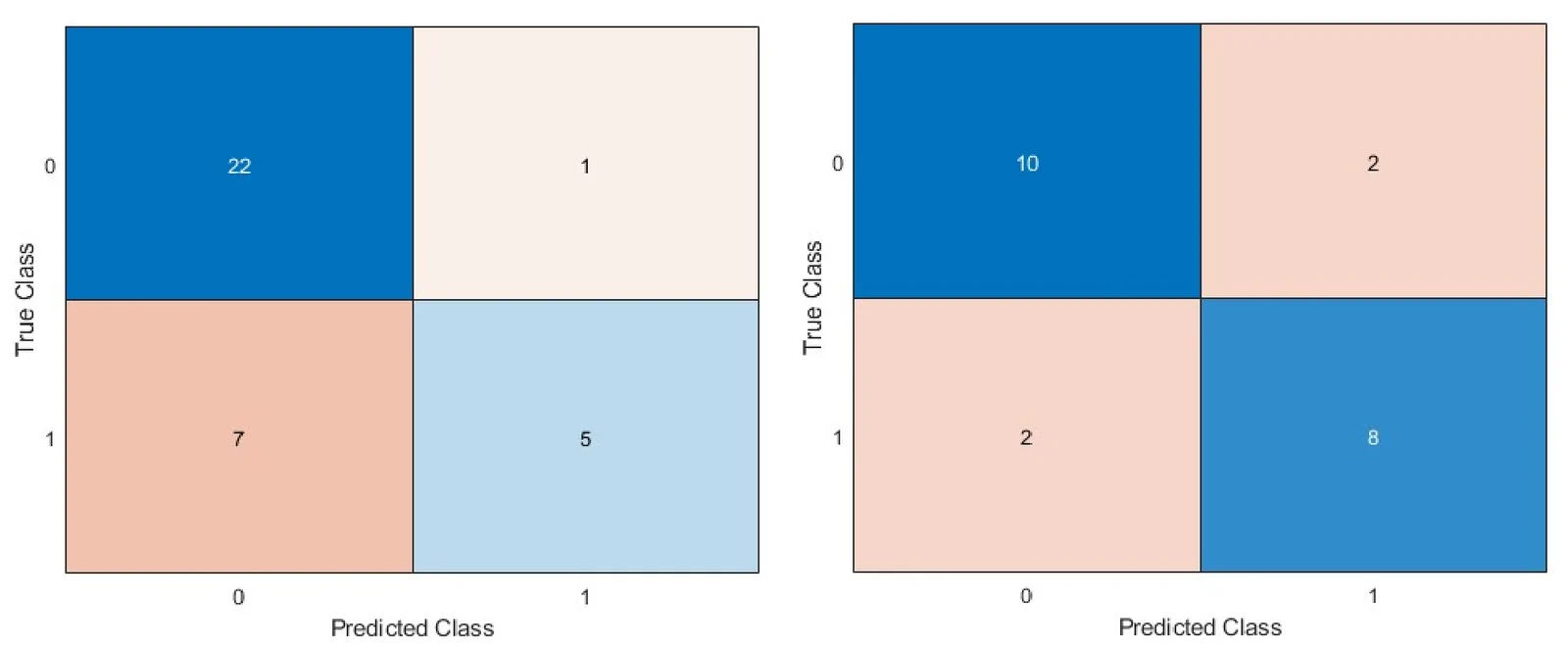
Predictive confusion precision of DLC in identifying entire sessions: 77.14% (50–50% data partition, 50 epochs, 35 sessions) and 81.82% (2/3-1/3, 150 epochs, 22 sessions) without data sorting; 74.29% and 77.27% using sorting.

Figure 16 shows confusion metrics for the DLC predicted labels output in the last 50% and 1/3 of the reserved data set in 35 and 22 sessions, respectively. DLC was more successful without using data sorting in padding (Figure 11), although its casual model misfires in prediction (falling in accuracy slightly above 50% in some runs). Data sorting improves the computing stability and adaptability of LSTM models, but at the expense of a notable drop in prediction accuracy.

**Figure 17:**
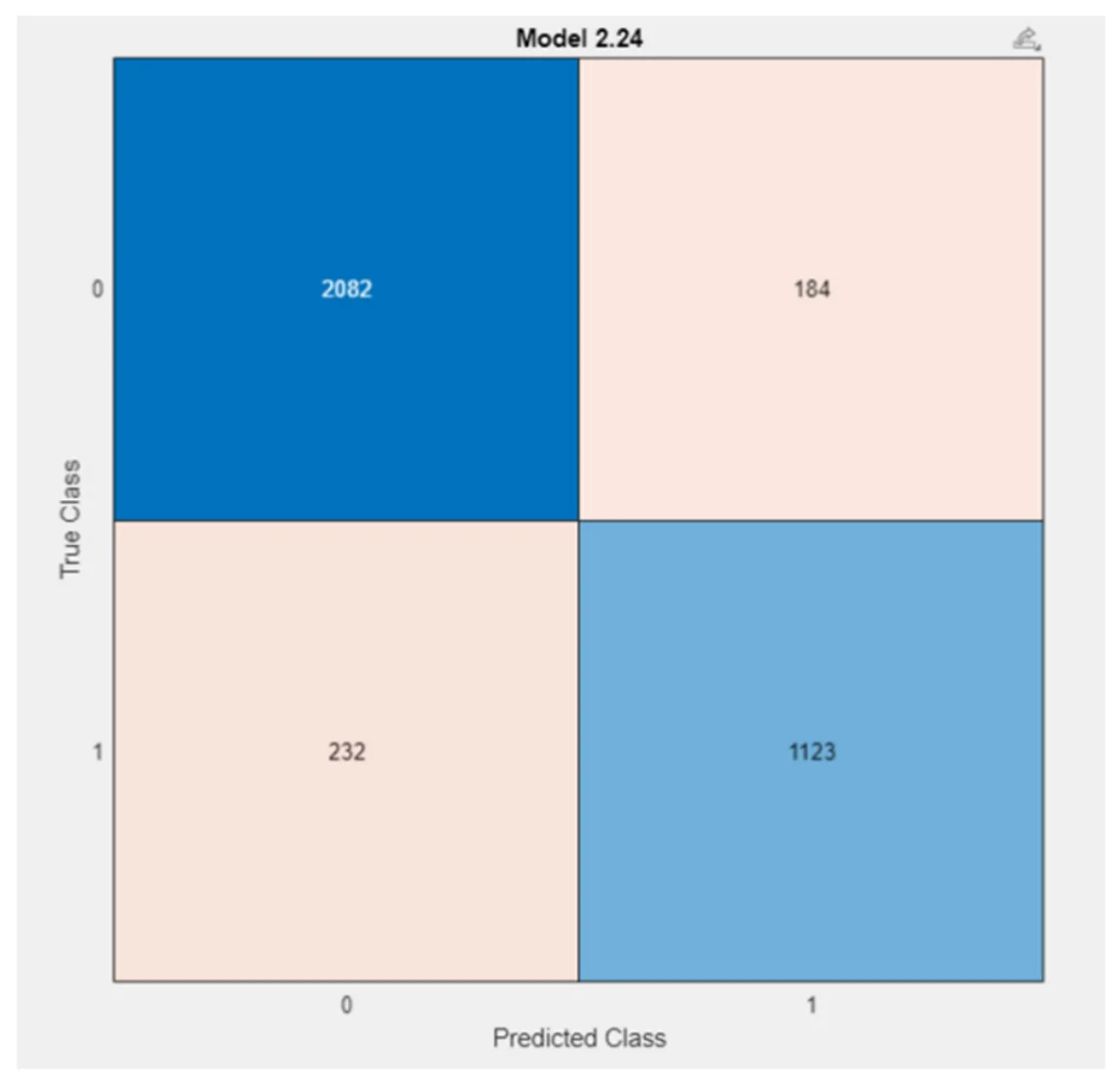
CLA validation confusion matrix of the EBT model for the first 3621 data rows: validation and prediction accuracy 88.5% and 66.1% (50–50% partition of the data), 5-fold cross-validation.

DLC models obtained validation accuracy (more than 90%) in training (Figure 12) analogous to those in the development of EBT (Figure 17), although not significantly implying the generalisability of EEG patterns (as in CLA). The optimal prediction model is able to select (stop the gradient adaptation) when detecting the loss (cross-entropy) minima in training progress, corresponding to the best matching generalisation on unknown data (as shown in Figure 12). The recurrent DLC strategy based on LSTM gate updates (Section 5.2) eliminates ambiguity in complex EEG signal dynamics better than the second-compared conventional classification row-to-row methodology (Section 4). However, LSTM time processing shows uncertainty in computing next-cell states, which induces the final DLC output.

Figure 17 illustrates the CLA confusion validation matrix of true–false EBT identifications using the 50% data in training. The lower generalisation of EBT models to predictive EEG patterns could result from higher training accuracy, which fundamentally implies overall performance. Uncontrolled tree growth based on the default leaf size can result in a deep binary branch structure. This problem could be partly solved by setting some limits on the growth of weak-tree learners with larger leaves, not losing predictive power, and controlling the minimal number of bagging observations per leaf in the ensemble setup. Most CLA models are fast in development, adaptable, and easy to interpret with an adequate real-time response (binary trees and their ensembles). They benefit from additional training, computing extensions, and algorithms in data splitting, improving their performance in solving problems (F. MATLAB—Classification Learner App (CLA) https://www.mathworks.com/help/stats/classificationlearner-app.html. Choose Classifier Options https://www.mathworks.com/help/stats/choose-a-classifier.html. Ensemble algorithms https://www.mathworks.com/help/stats/ensemble-algorithms.html (all accessed on 18 September 2026)).

**Figure 18:**
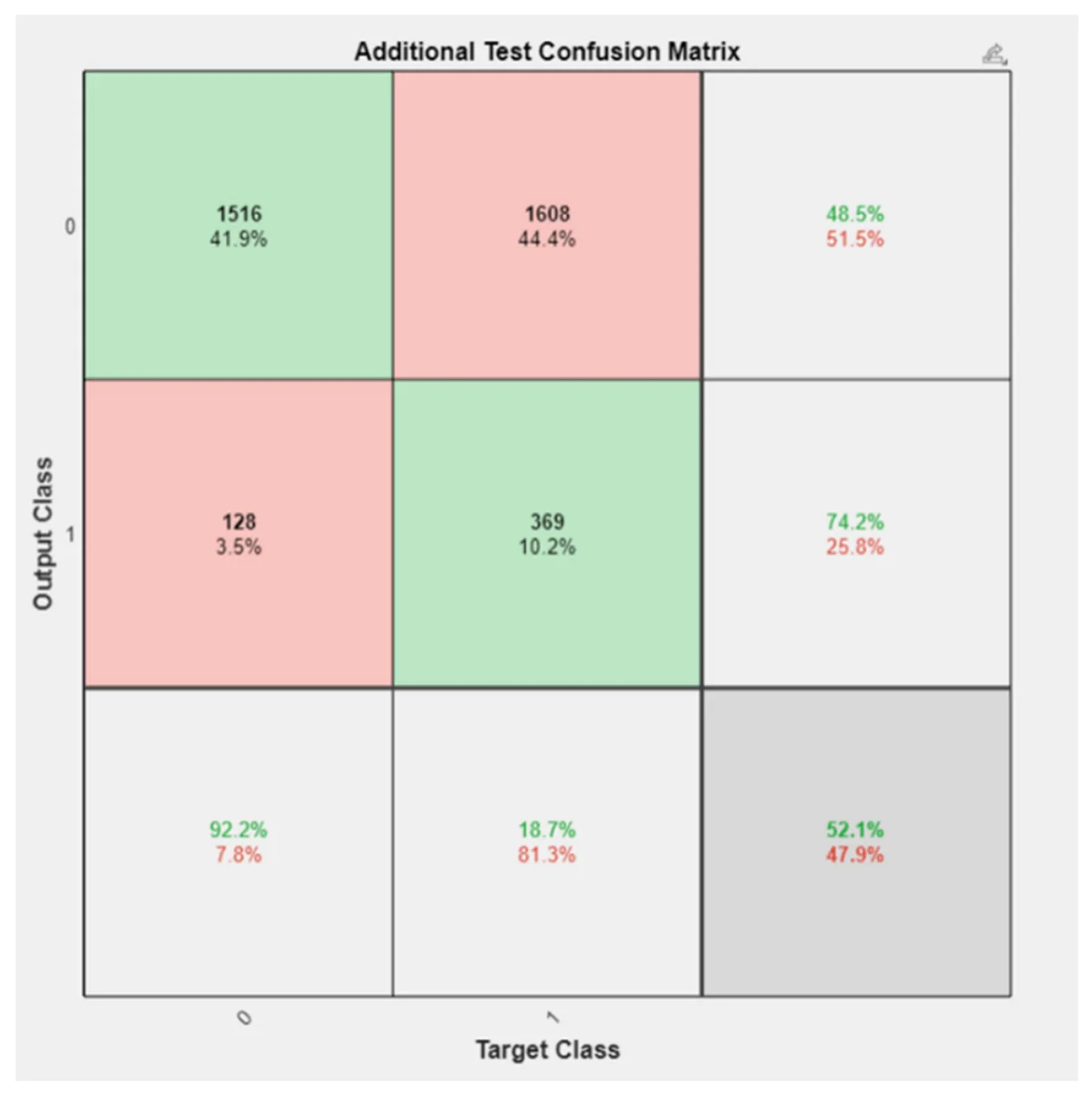
SNN test confusion metrics in prediction of the last 3621 data rows: prediction accuracy 52% (50–50% partition of the data); cross-entropy/error: training = 0.586/0.345, validation = 0.589/0.330.

Figure 18 displays the SNN prediction confusion matrix using the 50–50% partition of the data. As mentioned in Section 6, many methods could be found to improve the generalisation of the SNN in prediction pattern labels and void overfitting in training (early stopping, retraining, multiplication, regularisation, data division, indexing, etc.); see MATLAB supporting information (G. Shallow Neural Network (SNN) https://www.mathworks.com/help/deeplearning/ref/network.train.html. Improve Shallow Neural Network Generalisation and Avoid Overfitting https://www.mathworks.com/help/stats/fitcnet.html (all accessed on 18 September 2026)). The standard 2-layer feedforward SNN architecture (Figure 10) does not appear to be capable of obtaining higher predictive accuracy. Another reason could be the limited number of EEG data records used in these experiments; increasing the size of the training set and the input vector may partially solve the problem.

**Figure 19:**
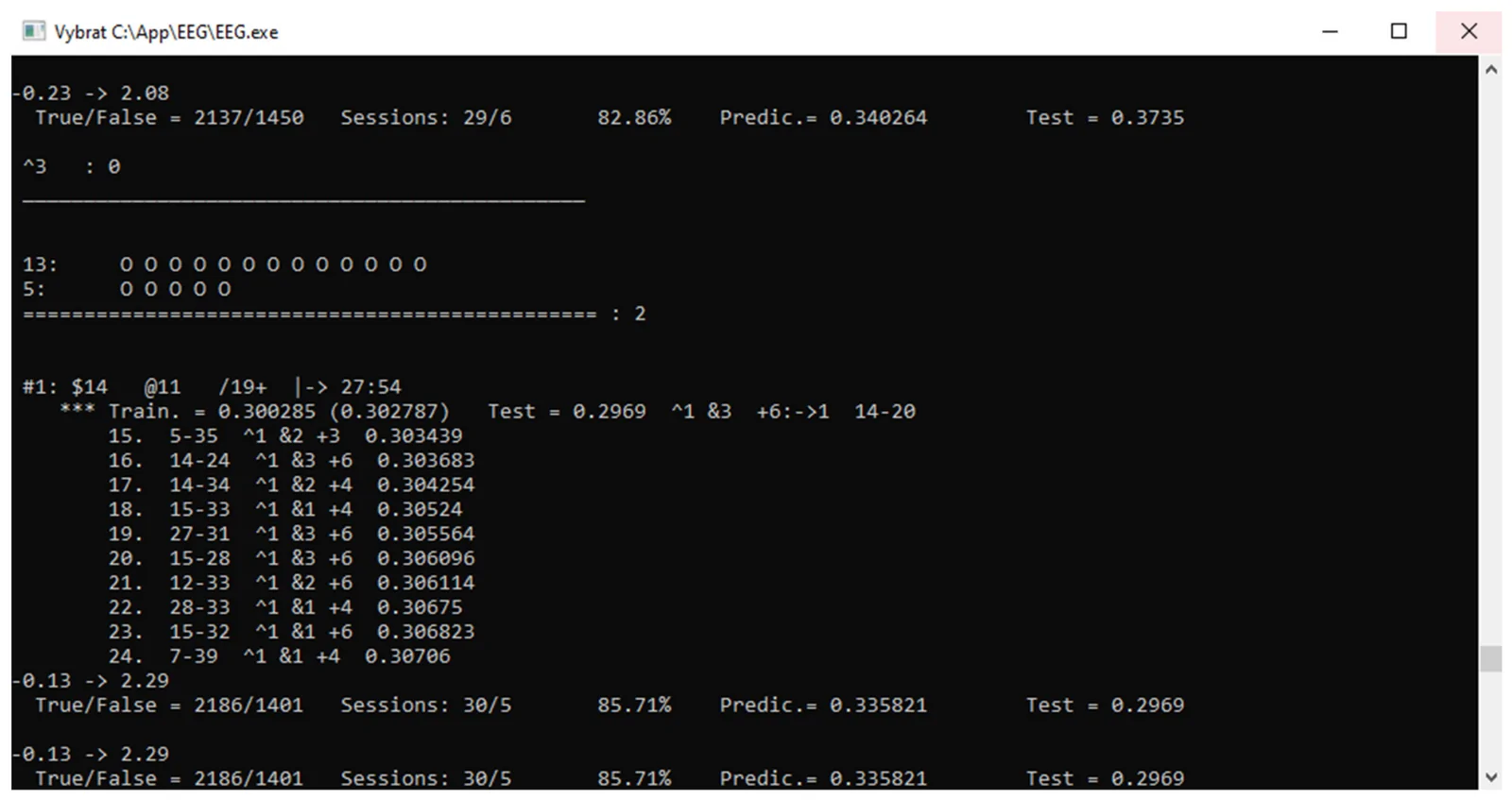
Demonstration of the DCN application software (H. DCN C++ parametric software with one merged Excel EEG data set: https://drive.google.com/drive/folders/1ZAw8KcvDEDM-i7ifVe_hDoS35nI64-Fh?usp=sharing. https://nextcloud.vsb.cz/s/EFDJ7mRyxfHkjSc (all accessed on 18 September 2026)) in mental state identification; the developed DBN structure consists of two layers, including 1st: 13 and 2nd: 5 selected block nodes producing optimal PDE rational or periodic transform components (accuracy = 85.71%, 50/50% training/test data set). The first layer ranked list of the best input node combination couples is displayed.

The list in Figure 19 in DCN execution ranks the most successful first-layer input couples (second column) in self-standing test error solutions (last column values), which are produced backward with respect to the only available first layer (^) by a newly added DCN node block: #14 in evaluating several types of PDE components (and 1–5) with specific numbers of 4–6 parameters (+). The best components are consequently undertaken for one of them to possibly be included in the extended overall sum model (to minimise output errors).

Table 6 resumes the predictive accuracy in true/false responses of the DLC, DCN, CLA, and SNN models based on the two evaluated methods: sequential entire session and data row computing strategies comparing various ML techniques. The predictive output of the DCN 1a models is statistically assigned to the highest percentage accuracy obtained in each data row separately, identifying the concentration label ‘0’/‘1’ throughout the processing session, while the recurrent LSTM gates update the internal memory frame at each time step to receive the final DLC output from the last computed state.

## 8. Discussion

Data pre-processing primarily affects the classification results in EEG concentration states. Rapid development of in-home headsets and accessories with easy-to-use benefits is evident. This compact equipment measures feature signals in several standardised EEG channels, allowing automatic built-in data transformations and analyses in pre-processing prior to recording or visualisation. However, the automatically supplied data features cannot cover the entire range of parameters and characteristics that are to be investigated in research experiments. The acquisition and analysis of large EEG data is challenging, but fundamental, for obtaining specific sets in the format applicable to ML. Casual fault classifications of DLC models result from uncertainty in sequentially updated multivalent memory states in iterative time steps with tidy variability in precision, which eventually falls below acceptable limits (<60%) in some misfire runs (E. MATLAB—Deep Learning neural network for Classification (DLC) https://www.mathworks.com/help/deeplearning/ug/deep-learning-in-matlab.html, www.mathworks.com/help/deeplearning/ug/classify-sequence-data-using-lstm-networks.html. Classify ECG Signals Using Long Short-Term Memory Networks https://www.mathworks.com/help/deeplearning/ug/classify-ecg-signals-using-long-short-term-memory-networks.html (all accessed on 18 September 2026)). This troubleshooting occurs naturally additionally due to greater unambiguity in the recurrent strategy, inadequate model initialisation, overfitting, or inadequacies in gradient adaptations captured in local error depressions.

Ambiguity and noise in EEG signals (measured by ordinary headband sensors, Figure 1) cause prevalent problems in adequate pattern modelling, despite using advanced ML methods. The data seem to comprise inapplicable apparent correlations that are not properly connected to the output labels or covered by essential input features. These problems result in partially defective models, which are certainly difficult to apply to new input in prediction. The first applied and evaluated methodology (Section 4) shows some restrictions and limitations in real-time EEG pattern detection, despite its higher predictive accuracy. Problems arise if the session start/end time interval is not exactly defined, i.e., it is unclear where to stop computing in the final class assessment in all sequenced series.

The time costs in forming DCN models are naturally higher than the elaborated AI techniques. DCN progressively evolves BT structures by selecting the optimal node inputs and applicable PDE components in the backward production process (facing combinatorial explosion in searching space-error minima). Model definition and self-optimisation adaptation algorithms should be further improved to eliminate limitations in learning capabilities and development time. The modular DfL concept implemented in DCN, based on the innovative double-wave signal representation of complex EEG dynamics in PDE solutions, outperforms standard CLA techniques. Practical implications for the one-session percentage evaluation are that dynamically evolved optimised tree structures, extended to the proposed coupled data processing, could further improve this platform-based and successful EEG modelling approach. Secondary frequency input is generally ignored in ML, except for complex-valued networks, which process bound pairs of input variables in a complex form (real and imaginary parts in complex arithmetic); however, their computing principles naturally do not differ from the routine fixed-structure functionality and error backpropagation of artificial NNs [31].

## 9. Conclusions

Two different types of methodologies and various computing approaches were compared in the above EEG self-concentration identification experiments. DCN and DLC using the first entire session sequence strategy, evaluating/computing one-class output for all included data rows, obtained the highest prediction accuracy. The DLC results can be improved if session data sorting (to reduce padding) is not applied, although negatively impacting the model computing stability and robustness (which could be further investigated). The recurrent LSTM cell-state time updates differ fundamentally from the second group of ML techniques in the conventional sequence-to-sequence output strategy. Processing periodic DCN signals in doubled amplitude and phase complex form proved its eligibility to model EEG patterns. DCN dynamical evolutionary BT structures produce modular PDE models by extending by the node blocks. Ensemble trees were found to be the most appropriate MATLAB modelling tool for the interpretation of mental stats in the secondary approach. The performance of EBT models could be partially improved by additional settings or algorithm extensions (according to the supporting documentation). Measurements of complex EEG data on a high-tech apparatus (rather than a home headband designed also for scientific purposes) can improve the findings and results in future investigation and experiments. However, these procedures usually produce row big data in a standardised form, which requires sophisticated pre-processing, transform, and feature extraction to have a predominant impact on the following modelling and classification experiments.

## Figures and Tables

**Table 1:** Base frequency distinct brain waves.

WAVE	FREQUENCY	STATE
Delta	below 4 Hz	Normal in moderate and deep sleep, dreamless, absence of physical activity and awareness, no sense and perception, deep meditation
Theta	4–8 Hz	Partial consciousness and perception, light sleep, deep relaxation, inward focussing, drowsiness, vivid dreaming, drowsy state
Alpha	9–13 Hz	Physical and emotional relaxation, light meditation or snoozing, passive attention, reflective and restful mind, symmetric wave amplitudes 40–100 µV by closed eyes
Beta	14–30 Hz	Normal activity, awareness and perceptiveness, medium-concentration activity, busy mind, anxiety, external attention
Gamma	above 30 Hz	Elevated mind, massive mental activity, problem solving, high levels of consciousness and spirits

**Table 2:** Muse 2 brain-sensing headband—technical features (A. Muse 3-channel EEG headset https://choosemuse.com/pages/science (accessed on 18 September 2026)).

Variable	Description	Range
RAW {TP9, AF7, AF8, TP10}	Raw brainwaves for each of the four sensors	0.0–1682.815 μV
AUX RIGHT	Raw brainwaves for the auxiliary USB sensor	0.0–1682.815 μV
HIS {TP9, AF7, AF8, TP10}	Data quality for each of the 4 sensors (HSI = Horseshoe Indicator)	1 = Good, 2 = Medium, 4 = Bad
Headbands On	Basic data quality indicator if the headband is on the head correctly	1 = True, 0 = False
Samplings Rate	Frequency	256 Hz
Noise Suppression	DRL–REF feedback	2 μV noise floor, 50 or 60 Hz notch filter
Input Range	Coupled signal	2 mV p-p AC

**Table 3:** Distribution of classes in the EEG data set (50–50% or 2/3-1/3 used in training–prediction).

Training–Prediction Data Partition/Classes	“0” Rows	“1” Rows	“0” and “1” Rows
50–50%	2266–1644	1355–1978	3621–3622
2/3-1/3	3147–763	1681–1652	4828–2415
100% of data	3910	3333	7243

**Table 4:** Understanding the binary confusion matrix.

Class	0	1
**0**	0-true	1-false
**1**	0-false	1-true

**Table 5:** **DCN** confusion predictive results: precision 70.0% (50–50% of the data) and 73.4% (2/3-1/3 data) for the last 3621 (50% data ‘0’: 1644 and ‘1’: 1977) and 2414 (1/3 ‘0’: 763, ‘1’: 1651) rows.

Class	0	1	Class	0	1
**0**	1053	499	**0**	548	427
**1**	591	1478	**1**	215	1224

**Table 6:** Summary of the predictive accuracy in mental state identification.

Order	Model	50–50% Accuracy	2/3-1/3 Accuracy	50–50% True/False	2/3-1/3 True/False	Identification Labels of
1a.	DCN (% eval.)	85.71	90.91	30/5	20/2	Entire sessions
2a.	DLC	77.14	81.81	27/8	18/4	Entire sessions
2b.	DLC (sorting)	74.29	77.27	26/9	17/5	Entire sessions
1b.	DCN	70.0	73.47	2531/1090	1772/642	Data rows
3.	CLA (EBT)	66.12	67.65	2393/1228	1631/783	Data rows
4.	SNN	52.15	54.81	1885/1736	1323/1091	Data rows

## Data Availability

The data presented in this paper and parametric C++ software are available for free. The Personal concentration data sets are available in the Kaggle public repository: https://www.kaggle.com/datasets/dqmonn/personal-eeg-tasks. The DCN C++ parametric software with one merged Excel EEG data set is available at: https://drive.google.com/drive/folders/1ZAw8KcvDEDM-i7ifVe_hDoS35nI64-Fh?usp=sharing and https://nextcloud.vsb.cz/s/EFDJ7mRyxfHkjSc (all accessed on 18 September 2026).
